# Hypoxia‐preconditioned adipose‐derived stem cells with injectable small intestinal submucosa for enhanced cartilage repair in osteoarthritis

**DOI:** 10.1002/btm2.70116

**Published:** 2026-02-02

**Authors:** Kun Yu, Liang Ma, Pengkun Han, Yinshen Liu, Longfei Zou, Sen Wang, Jiesi Hu, Kai Zhong, Jiaqiang Liu, Bo Guo, Jie Zou, Houyin Shi, Xing Guo, Meiyun Tan

**Affiliations:** ^1^ Department of Orthopedics Affiliated Hospital of Southwest Medical University Luzhou China; ^2^ Department of Orthopedics The Affiliated Hospital of Traditional Chinese Medicine of Southwest Medical University Luzhou China; ^3^ Day Surgery Center Affiliated Hospital of Southwest Medical University Luzhou China

**Keywords:** adipose‐derived mesenchymal stem cells, cartilage repair, hypoxia preconditioning, injectable SIS, osteoarthritis

## Abstract

Osteoarthritis (OA) is a widespread degenerative condition marked by inflammation‐induced damage to chondrocytes and gradual breakdown of the cartilage extracellular matrix. Adipose‐derived mesenchymal stem cells (ADSCs) hold potential for treating OA due to their capacity to differentiate into various cell types and their paracrine signaling functions. However, the inflammatory environment in OA reduces ADSC viability post‐injection, while the absence of a supportive carrier causes significant cell loss, impairing their capacity for cartilage repair. To address these challenges, we improved the stemness and paracrine activity of ADSCs through hypoxia preconditioning and integrated them into an injectable small intestinal submucosa (SIS) tissue repair scaffold. This resulted in an SIS + ADSC composite material, designed for intra‐articular injection to enhance cartilage repair in arthritis. Our findings revealed that exposing ADSCs to 2% oxygen during hypoxia preconditioning and incorporating them into injectable SIS significantly increased the secretion of growth factors (VEGF, bFGF, EGF) and upregulated key hypoxia and stem cell markers (HIF‐1α, NANOG, SOX‐2, Oct‐4). In a rat OA model, hypoxia‐preconditioned SIS + ADSC composites markedly enhanced cartilage repair by stimulating anabolic activity, suppressing catabolic pathways, and reducing inflammation, thereby exhibiting strong protective and reparative effects. In summary, combining hypoxia preconditioning with injectable SIS offers an innovative and effective approach to optimize OA treatment by enhancing paracrine signaling, paving the way for new insights and technologies in cartilage repair within regenerative medicine.

## Graphical abstract

Schematic illustrating the preparation of the SIS + ADSCs(H) composite and its potential for cartilage repair in OA. Hypoxia‐preconditioned ADSCs, combined with injectable SIS, exert protective and reparative effects on chondrocytes by enhancing paracrine activity, promoting anabolic processes, inhibiting catabolic processes, and suppressing inflammation.

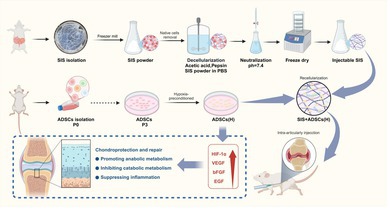

**Translational Impact Statement**

This study introduces a novel method for osteoarthritis (OA) treatment by improving the therapeutic potential of adipose‐derived mesenchymal stem cells (ADSCs). By using hypoxia preconditioning and embedding ADSCs in an injectable small intestinal submucosa (SIS) scaffold, the composite material greatly enhanced cartilage repair in OA. This approach optimizes ADSC function, boosts paracrine activity, and promotes cartilage regeneration, presenting a promising clinical strategy that could revolutionize OA therapy and joint health regeneration.

## 1 INTRODUCTION

Osteoarthritis (OA) is a prevalent chronic joint disorder involving multiple structural changes, such as degeneration in articular cartilage, subchondral bone, ligaments, joint capsules, synovium, and periarticular muscles. OA is a widespread and disabling condition, with its incidence growing worldwide and contributing to an increasing global health burden. The knee joint is the most frequently affected area, contributing to around 85% of the global OA burden.^1^ Knee OA progression is closely linked to the loss of articular cartilage integrity. Cartilage injury and repair processes often involve matrix degradation and pro‐inflammatory responses, which are key contributors to the disease's development.^2^ Due to its avascular, aneural, and alymphatic nature, articular cartilage has minimal self‐repair ability following injury.^3^ Traditional OA treatments encompass non‐pharmacological strategies like education, self‐management, and lifestyle changes, alongside pharmacological options including NSAIDs, opioids, topical analgesics, corticosteroids, and hyaluronic acid injections.^4^, ^5^ However, these approaches generally offer temporary symptom relief without preventing cartilage damage or slowing arthritis progression.^6^, ^7^ Intra‐articular injections are currently the main non‐surgical treatment for knee OA, involving agents like corticosteroids, hyaluronic acid, medical chitosan, and platelet‐rich plasma (PRP). However, these treatments are often limited by side effects and inconsistent efficacy. Corticosteroids may worsen joint degeneration, the pain‐relief effects of hyaluronic acid are debated, and PRP outcomes are dose‐dependent, potentially reducing efficacy and increasing adverse effects.^8^, ^9^, ^10^, ^11^ In summary, existing non‐surgical treatments fail to meet clinical demands, allowing OA to advance and often leading to the need for surgical intervention.^12^ Thus, there is a pressing need for more effective and safer therapeutic strategies.

In recent years, stem cell‐based therapies have emerged as promising strategies in tissue engineering, demonstrating positive outcomes in both animal models and clinical trials for articular cartilage repair.^13^, ^14^, ^15^, ^16^, ^17^, ^18^, ^19^ Among these approaches, adipose‐derived mesenchymal stem cells (ADSCs), a subset of mesenchymal stem cells (MSCs), have shown distinct advantages in cartilage repair, supporting their growing clinical application.^20^ While bone marrow‐derived MSCs are widely used in clinical practice, they have limitations: they are unsuitable for patients with hematological or bone marrow disorders, require multiple punctures to obtain sufficient quantities, and their efficiency declines when contaminated by peripheral blood. MSCs derived from perinatal tissues, such as the placenta, amniotic membrane, and umbilical cord, have higher activity and greater differentiation potential but are allogeneic, which carries a risk of disease transmission.^21^, ^22^, ^23^ Compared with stem cells derived from bone marrow or perinatal tissues, ADSCs are more accessible, widely available, involve minimal surgical trauma, and are cost‐effective.^24^, ^25^ Therefore, ADSCs offer superior clinical potential compared with other MSCs. However, the inflammatory microenvironment in OA compromises ADSC viability, inhibits extracellular matrix synthesis, and impairs cell function, ultimately reducing their therapeutic effectiveness for OA.^26^ Therefore, there is a need to develop simple and practical strategies to enhance the repair capacity of stem cells.

Hypoxia preconditioning is a widely recognized and effective strategy for improving the repair potential of stem cells.^27^ Research indicates that short‐term hypoxic exposure enhances MSC survival, reduces apoptosis, and stimulates the production of growth factors. Additionally, hypoxia preconditioning has been found to boost the therapeutic efficacy of MSCs in treating conditions including cerebral ischemia and femoral head necrosis.^28^, ^29^ Notably, ADSCs can adapt to significantly lower oxygen levels (1%–5%) compared with other cell types (20%–21%).^30^ This characteristic enables ADSCs to demonstrate superior adaptability in hypoxic conditions. Hypoxia preconditioning further amplifies their stem cell activity, paracrine signaling, proliferation, and survival rates.^31^, ^32^, ^33^, ^34^ Consequently, ADSCs can survive more effectively in the hypoxic environments of the knee joint cavity and articular cartilage, secreting higher levels of paracrine therapeutic factors.^35^, ^36^

**FIGURE 1:**
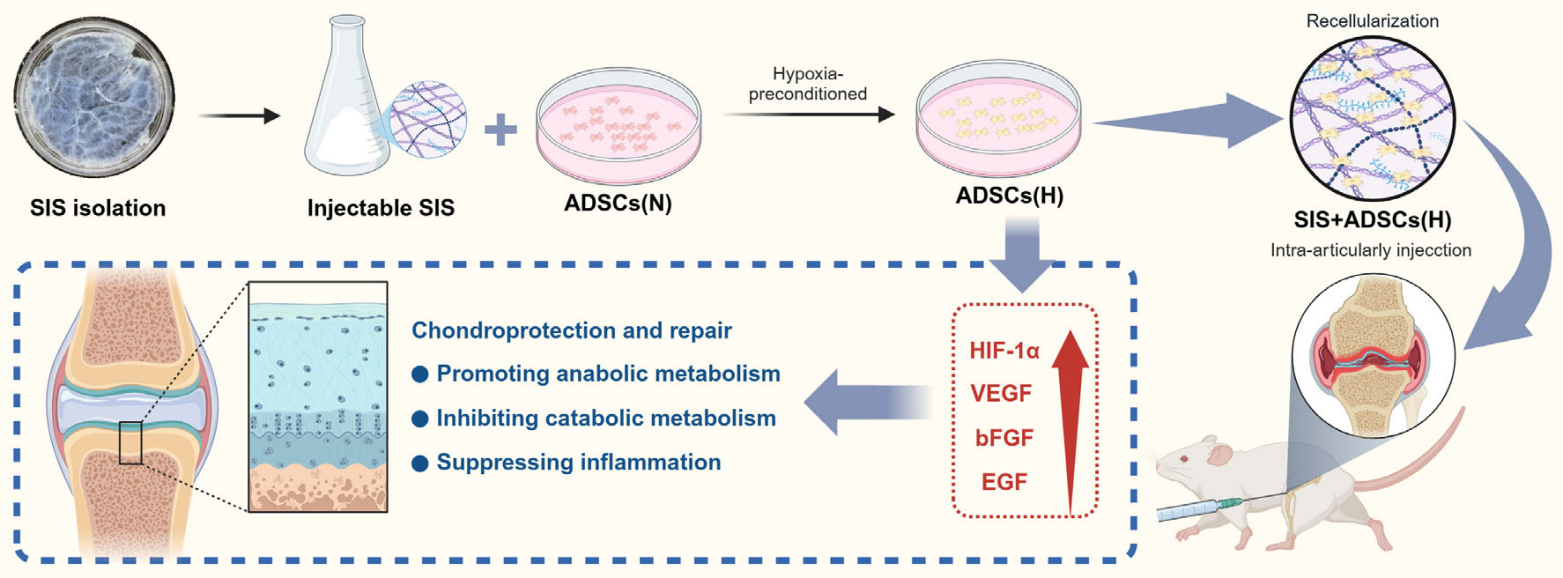
The schematic diagram shows the successful preparation of SIS + ADSC composite using hypoxia pretreated adipose derived stem cells (ADSCs) as cell source and injectable SIS as scaffold. It was injected into the knee joint cavity of rats to protect and repair chondrocytes.

Traditional two‐dimensional cell culture frequently causes contact inhibition or dedifferentiation, while direct transplantation of cells to injury sites often results in stem cell loss due to the absence of carrier support.^37^ Current approaches to tissue injury repair rely on the development of biological scaffolds, which mimic the active and passive mechanical properties of organs and are combined with stem cells to promote tissue regeneration.^38^, ^39^, ^40^ Small intestinal submucosa (SIS) is a natural acellular extracellular matrix derived from porcine small intestine. It is rich in collagen, glycosaminoglycans, fibronectin, hyaluronic acid, and cytokines. With its outstanding biodegradability, biocompatibility, and capacity to promote cell adhesion, proliferation, and migration, SIS is regarded as an ideal scaffold for tissue repair. Researchers have utilized MSC‐loaded membrane‐like SIS patches for transplantation to injury sites, promoting regeneration and repair in tissues like the urethra, skin, heart, bone, and tendons.^38^, ^39^, ^40^, ^41^, ^42^, ^43^, ^44^ However, this transplantation approach is invasive and technically challenging. Recent studies reveal that combining MSCs with injectable SIS delivers favorable therapeutic results in repairing damaged tissues, such as the vocal cords and heart.^45^, ^46^ This composite material enables precise, rapid, and minimally invasive delivery to injury sites via injection, significantly improving convenience. Thus, we developed a novel biomaterial composite by integrating hypoxia‐preconditioned ADSCs with injectable SIS, providing a new method for non‐surgical arthritis treatment. However, it is unclear whether hypoxia preconditioning enhances the cartilage repair capacity of ADSCs in OA. To address this, we utilized ADSCs as the cellular source and injectable SIS as the scaffold to develop the SIS + ADSC composite. We then evaluated the impact of hypoxia preconditioning on its cartilage repair potential in a rat knee OA model(Figure 1).

## 2 MATERIALS AND METHODS

### 2.1 Isolation and culture of rat ADSCs

Rat ADSCs were isolated using the collagenase digestion method as described in previous studies.^47^ The rats were anesthetized using a 3% pentobarbital solution, and the inguinal adipose tissue was carefully harvested and placed into a petri dish with phosphate‐buffered saline (PBS). Blood, vessels, and fascia were precisely excised, followed by washing the tissue 3–5 times in PBS. The cleaned adipose tissue was minced into a paste, placed in a 50 mL centrifuge tube, and digested with 0.2% type II collagenase in a 37°C water bath for 1 h. Upon completion of digestion, the process was halted by adding complete cell culture medium. After filtering the adipose suspension through a 70 μm cell strainer, it was centrifuged at 1500 rpm for 10 min, and the supernatant was discarded. The pellet was resuspended in PBS and centrifuged again at 1000 rpm for 5 min, followed by discarding the supernatant. The pellet was then cultured in DMEM/F12 medium supplemented with 10% FBS at 37°C in a 5% CO_2_ atmosphere to isolate ADSCs. After 24 h, non‐adherent cells were removed by washing the culture plates with PBS. The adherent cells were further cultured for 1 week, with the medium replaced every 2–3 days to obtain ADSCs.

### 2.2 Identification of rat ADSCs

#### 2.2.1 Flow cytometry analysis

ADSCs were suspended in PBS at a concentration of 1 × 10^6^ cells/mL and incubated with antibodies including CD29‐FITC, CD34‐PE, CD44‐PE, CD90‐PECY7, and CD45‐A647.^19^ After being incubated at room temperature for 30 min, the cell suspensions underwent centrifugation at 2000 rpm for 5 min. The supernatant was discarded, and the cell pellet was resuspended in 100 μL PBS for flow cytometry analysis using a BD FACSAria system (New Jersey, USA).

#### 2.2.2 Osteogenic differentiation

Cells were grown in 6‐well plates until reaching 70%–80% confluence, after which osteogenic induction medium (Pricella, China) was added. The medium was replaced every 3 days, and after 28 days of induction, the presence of calcified extracellular matrix was evaluated using Alizarin Red S staining solution (Pricella, China).

#### 2.2.3 Adipogenic differentiation

Cells were cultured in 6‐well plates to 100% confluence and then incubated with adipogenic induction medium (OriCell, China), with medium changes performed every 1–3 days. Following 21 days of induction, Oil Red O staining (OriCell, China) was employed to detect and visualize lipid vacuoles.

#### 2.2.4 Chondrogenic differentiation

Cells were grown in 15 mL centrifuge tubes, and once cell clusters formed, the tubes were gently tapped to dislodge the chondrospheres from the bottom, allowing them to remain suspended in the medium. The chondrogenic induction medium (OriCell, China) was refreshed every 2–3 days. The induction process was continued until chondrospheres of the desired size were formed. The chondrospheres were sectioned and stained with Alcian Blue (OriCell, China) to detect acidic mucopolysaccharides in the cartilage tissue.

### 2.3 Preparation of SIS + ADSC composite materials

SIS was prepared following previously described methods.^45^, ^48^ Briefly, fresh porcine small intestine tissue was harvested from market pigs (around 100 kg at 6 months) within 3 h of sacrifice. The muscular layer, mucosa, and serosa were mechanically removed, and the small intestines were thoroughly cleaned with saline to isolate SIS from the porcine jejunum. The cleaned tissue was freeze‐dried at −80°C for 48 h using a freeze dryer (Labconco, USA). The dried SIS was ground into 10–20 μm particles using a grinder (Jinxin, China) at −198°C. The SIS powder was stirred in a solution of 3% acetic acid and 0.1% pepsin for 48 h, followed by neutralization with sodium bicarbonate. The neutralized solution was freeze‐dried to produce the final SIS powder, which was sterilized with ethylene oxide (EO) gas. The SIS suspension was diluted in complete cell culture medium to prepare a 10 wt% SIS culture medium. When ADSCs in 10 cm culture dishes reached 70%–80% confluence, replace the original culture medium with a medium containing SIS. The SIS + ADSC composites were categorized into two groups: (1) the SIS + ADSC (N) group, cultured for 24 h under normoxic conditions (21% O_2_, 5% CO_2_) in an incubator (Thermo, USA); and (2) the SIS + ADSC (H) group, cultured for 24 h under hypoxic conditions (2% O_2_, 5% CO_2_) in an incubator (Thermo, USA).

### 2.4 Characterization of SIS + ADSC composites

#### 2.4.1 Cell viability

The SIS + ADSC composites were stained with a live/dead cell staining kit (Abbkine, China) by incubating them in the dark for 30 min, followed by two PBS washes and observation under an inverted fluorescence microscope (ECLIPSE Ti2‐U, Nikon, Japan).

#### 2.4.2 Scanning electron microscopy (SEM) observation

The SIS + ADSC composites were preserved with 2.5% glutaraldehyde (Sigma, USA) at 4°C for 24 h, dehydrated through a gradient ethanol series, freeze‐dried at −80°C for 24 h, vacuum‐dried overnight, coated with a gold layer, and examined under a scanning electron microscope (JSM‐IT700HR, JEOL, Japan).

#### 2.4.3 CM‐Dil labeling of ADSCs

ADSCs were labeled with CM‐Dil, mixed with SIS, incubated in the dark for 24 h, and analyzed using an inverted fluorescence microscope (ECLIPSE Ti2‐U, Nikon, Japan).

### 2.5 Preparation of primary chondrocytes

Primary chondrocytes were obtained from allogeneic male SD rats following previously reported methods.^47^ Briefly, knee joint cartilage tissues were collected and cut into small fragments. The cartilage fragments were digested with 0.25% trypsin at 37°C for 25 min, followed by digestion with 0.2% collagenase II at 37°C for an additional 2 h. The digestion process was stopped by adding complete cell culture medium. The suspension was passed through a 70 μm cell strainer, and chondrocytes were collected by centrifugation at 1200 rpm for 10 min, after which the supernatant was discarded. The resulting pellet was resuspended in PBS and centrifuged at 1000 rpm for 5 min, after which the supernatant was discarded. Chondrocytes were grown in DMEM/F12 medium containing 10% FBS. Third‐passage chondrocytes were selected for subsequent experiments.

### 2.6 Preparation of conditioned medium (CM)

The conditioned medium (CM) from SIS + ADSC composites and chondrocytes was prepared for in vitro experiments.^47^ Briefly, ADSCs and chondrocytes were plated at a concentration of 1 × 10^6^ cells per 10 cm dish in complete growth medium. Once ADSCs reached 70% confluence, the medium was replaced with 10 wt% SIS‐supplemented complete culture medium, and the cells were incubated under either normoxic or hypoxic conditions for 24 h. When chondrocytes reached 70% confluence, the medium was replaced with either 10 wt% SIS‐supplemented complete culture medium or fresh complete culture medium, and the cells were incubated under normoxic conditions for 24 h. The conditioned medium was harvested, then subjected to centrifugation at 4000 rpm and 4°C for 10 min, followed by filtration through a 0.22 μm cell filter. The resulting chondrocyte‐CM, SIS + chondrocyte‐CM, SIS + ADSC (Normoxia)‐CM, and SIS + ADSC (Hypoxia)‐CM were stored at −80°C for subsequent experiments.

### 2.7 Real‐time polymerase chain reaction (RT‐PCR)

RNA was extracted from each group (*n* = 3) using TRIZOL reagent (Thermo Fisher, USA) and subsequently reverse‐transcribed into cDNA with an RNA PCR kit (Life Technologies, USA). The RT‐PCR was carried out under the following conditions: an initial denaturation at 95°C for 2 min, followed by 50 cycles of 95°C for 15 s and 60°C for 30 s. Gene expression levels were analyzed using the ΔΔCT method. Specifically, the average CT value was normalized to the β‐actin internal control, and the difference was calculated as ΔCT. Relative gene expression was then determined by the formula 2^−ΔΔCT^. PCR primers (Additional file 1) were designed using PrimerBank software.

### 2.8 Enzyme‐linked immunosorbent assay (ELISA)

SIS + ADSC (N)‐CM and SIS + ADSC (H)‐CM (*n* = 3) were collected, and levels of epidermal growth factor (EGF), vascular endothelial growth factor (VEGF), and basic fibroblast growth factor (bFGF) were quantified using ELISA kits (Ray Biotech, USA) following the manufacturer's protocols.

### 2.9 Cell experiments

Chondrocytes were assigned to five groups: control group, IL‐1β group, SIS + chondrocyte‐CM group, SIS + ADSC (N)‐CM group, and SIS + ADSC (H)‐CM group. The IL‐1β group, SIS + chondrocyte‐CM group, SIS + ADSC (N)‐CM group, and SIS + ADSC (H)‐CM group were modeled by treating chondrocytes with IL‐1β (10 ng/mL) for 24 h. After IL‐1β treatment, the SIS + chondrocyte‐CM group, SIS + ADSC (N)‐CM group, and SIS + ADSC (H)‐CM group were treated with SIS + chondrocyte‐CM, SIS + ADSC (N)‐CM, and SIS + ADSC (H)‐CM, respectively, for 48 h. Meanwhile, the control group and IL‐1β group received chondrocyte‐CM treatment for 48 h.

#### 2.9.1 Immunofluorescence staining

Cultured chondrocytes were rinsed three times with pre‐cooled PBS, fixed in 4% paraformaldehyde for 15 min, and blocked with 3% BSA for 1 h. Cells were incubated overnight at 4°C with primary antibodies, including rabbit anti‐Col2a (dilution 1:200; Proteintech, China) and rabbit anti‐MMP13 (dilution 1:200; Proteintech, China). The cells were then incubated with a goat anti‐rabbit secondary antibody (CoraLite488 Goat Anti‐Rabbit IgG, dilution 1:200; Proteintech, China) at room temperature for 1 h. Cell nuclei were stained with DAPI (dilution 1:500; Beyotime, China) for 5 min. The slides were washed, and fluorescence images were acquired using an inverted fluorescence microscope (ECLIPSE Ti2‐U, Nikon, Japan). The average immunofluorescence intensity for each group was quantified using Image J software.

#### 2.9.2 Western blotting

Total protein was extracted from chondrocytes on ice for 30 min using a lysis buffer supplemented with phosphatase and protease inhibitors (Bimake, Houston, Texas, USA). Primary antibodies (anti‐Aggrecan, Abcam, UK; MMP13, Proteintech, China; anti‐ADAMTS5, Bioss, China; anti‐IL‐1β, Abcam, UK; β‐actin, Beyotime, China) targeting ACAN, MMP13, ADAMTS5, IL‐1β, and β‐actin were applied and incubated overnight at 4°C. The samples were incubated with HRP‐conjugated secondary antibodies (HRP goat anti‐rabbit IgG H&L, Beyotime, China; HRP goat anti‐mouse IgG H&L, Beyotime, China) at room temperature for 1 h. Immunoreactive bands were visualized using an enhanced chemiluminescence (ECL) Western blot detection kit (EpiZyme, China). Protein bands were detected using an imaging system (Fusion Solo 4, Connecticut, France). Band intensities were analyzed and visualized using Image J software.

### 2.10 Animal experiments

All animal experiments followed national guidelines and received approval from the Animal Care and Use Committee of Southwest Medical University before starting cell and tissue experiments (Approval No. 20221104‐015). Male Sprague Dawley (SD) rats (6 weeks old, 200 ± 20 g, *n* = 50) were obtained from the Animal Research Institute of Southwest Medical University and used to establish an osteoarthritis (OA) model.

The rats were kept in pathogen‐free environments with a 12‐h light/dark cycle and had unlimited access to food and water. To evaluate the in vivo effects of SIS and compare ADSC (N) with ADSC (H), 50 rats were randomly assigned to five groups: (1) normal control (NC); (2) OA model (OA); (3) SIS‐treated model (SIS); (4) SIS + ADSC (N)‐treated model (SIS + ADSC (N)); and (5) SIS + ADSC (H)‐treated model (SIS + ADSC (H)), with 10 rats per group. Seven‐week‐old rats were anesthetized with isoflurane and received a single 50 μL intra‐articular injection of 30 mg/mL monoiodoacetate (MIA) to induce an osteoarthritis (OA) model. They then underwent a one‐week recovery period.^47^ The SIS suspension was prepared by dispersing SIS in PBS to achieve a 10 wt% concentration. ADSCs preconditioned under normoxic or hypoxic conditions for 24 h were combined with 10 wt% SIS at a density of 1 × 10^6^ cells per mL to create injectable biomaterials SIS + ADSC (N) and SIS + ADSC (H). Following the 1‐week recovery period post‐OA modeling, rats in the NC and OA groups received 50 μL intra‐articular saline injections. Rats in the SIS, SIS + ADSC (N), and SIS + ADSC (H) groups were administered 50 μL intra‐articular injections of 10 wt% SIS, SIS + ADSC (N), and SIS + ADSC (H), respectively. All treatments were administered weekly on the first day of the week for a total of 4 weeks.^19^ All rats in this research were euthanized by cervical dislocation at the end of the trial. The work has been reported in line with the ARRIVE guidelines 2.0.

#### 2.10.1 X‐ray and Micro‐CT imaging

After 4 weeks of treatment, the rat knee joints were collected, and soft tissues, including muscles and skin, were removed. The remaining joint tissues were fixed in 4% paraformaldehyde. The x‐ray imaging of the knee joints was performed to evaluate and quantify OA severity. Micro‐CT scans were conducted using the SkyScan1276 system (Bruke, Germany) with a resolution of 17.4 μm/pixel. Three‐dimensional reconstructed images were generated using NRECON software. The main parameters examined were the volume of osteophytes, the ratio of bone volume to tissue volume (BV/TV), the number of trabeculae (Tb.N), the thickness of trabeculae (Tb.Th), and the separation of trabeculae (Tb.Sp).

#### 2.10.2 Histopathological analysis

Cartilage sections were stained with hematoxylin and eosin (HE) and safranin O‐fast green. A digital slide scanner (KFBIO, Jiangfeng Bio, China) was utilized to assess cartilage degeneration following the OARSI guidelines.^49^ For immunohistochemistry, sections were incubated overnight at 4°C in a humidified chamber with primary antibodies targeting Col2a, MMP13, and IL‐1β (dilution 1:200; Bioss, China). After incubating with the primary antibody, sections were treated with HRP‐linked secondary antibodies (dilution 1:200; Bioss, China) at 37°C for an hour. Immunoreactivity for Col2a, MMP13, and IL‐1β was semi‐quantified using Image J software. Expression of MMP13 and IL‐1β was quantified by counting the number of positive cells, while Col2a expression was assessed by measuring the positive‐staining area. The ultimate findings were presented as the percentage of antigen‐positive area or cell count in relation to the total area or overall cell count within the examined field of view.

### 2.11 Statistical analysis

Quantitative data were displayed as mean ± SEM, with statistical analysis carried out using GraphPad Prism 10.0 software (GraphPad Software, USA). All variables were assessed for normal distribution. For data that follow a normal distribution, Student's *t*‐test was used for comparing two groups, and one‐way ANOVA was employed for comparisons among several groups. The Kruskal–Wallis test was used for data that did not follow a normal distribution. A *p*‐value of <0.05 was considered statistically significant.

## 3 RESULTS

### 3.1 Characterization of ADSCs

**FIGURE 2:**
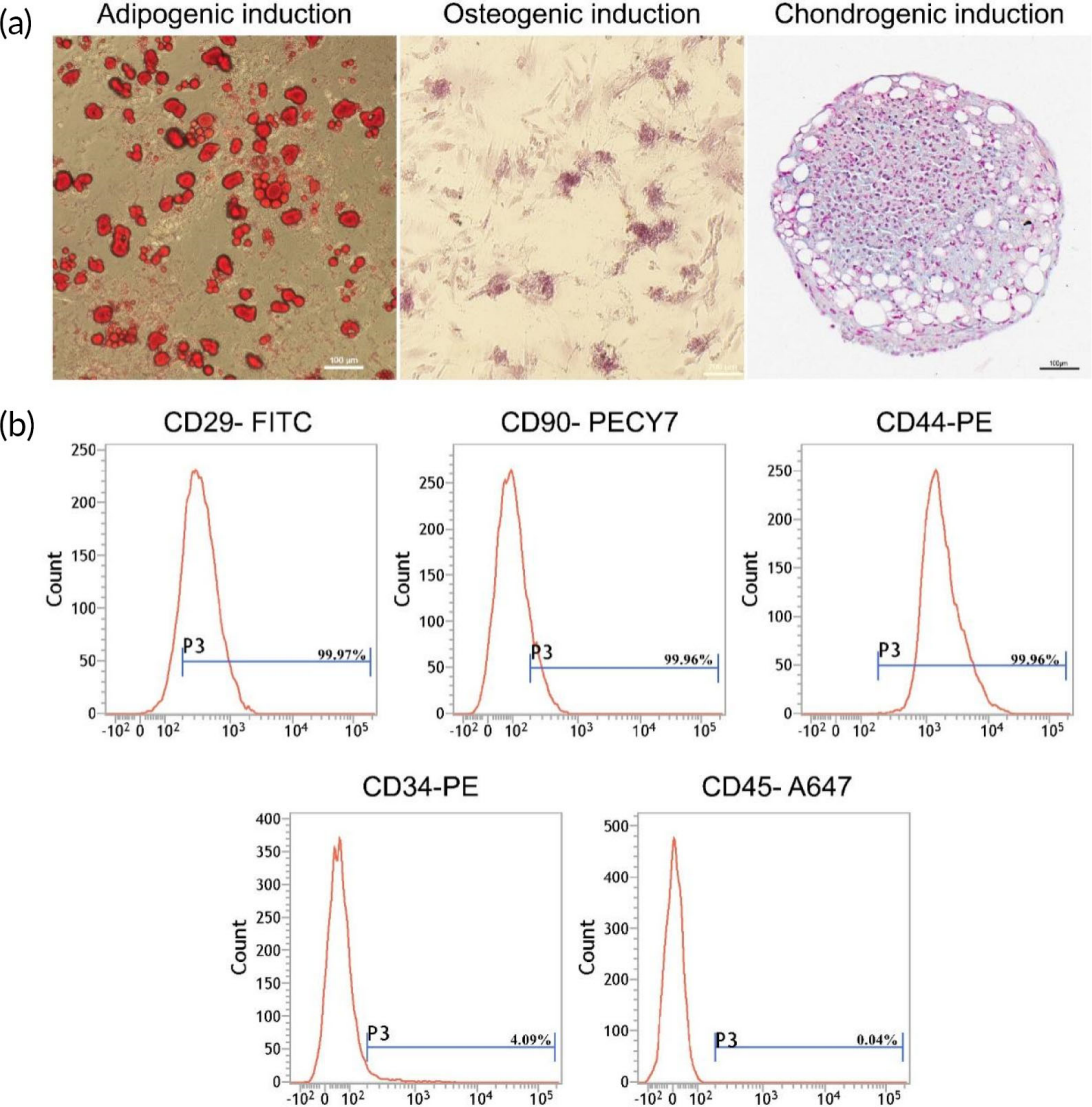
Characterization of Rat ADSCs. (a) Representative images of adipogenic‐induced (Scale bar, 100 μm), osteogenic‐induced (Scale bar, 200 μm) and chondrogenic‐induced (Scale bar = 100 μm) ADSCs. (b) Flow cytometry analysis of ADSCs.

As shown in Figure 2b, flow cytometry analysis demonstrated that cultured ADSCs expressed CD29, CD44, and CD90 at high levels (>95%) while showing low expression of CD34 and CD45 (<5%). Following in vitro induction, ADSCs successfully differentiated into osteogenic, adipogenic, and chondrogenic lineages, as confirmed by positive staining with Alizarin Red, Oil Red O, and Alcian Blue, respectively (Figure 2a).

### 3.2 Characterization of SIS + ADSC composites

**FIGURE 3:**
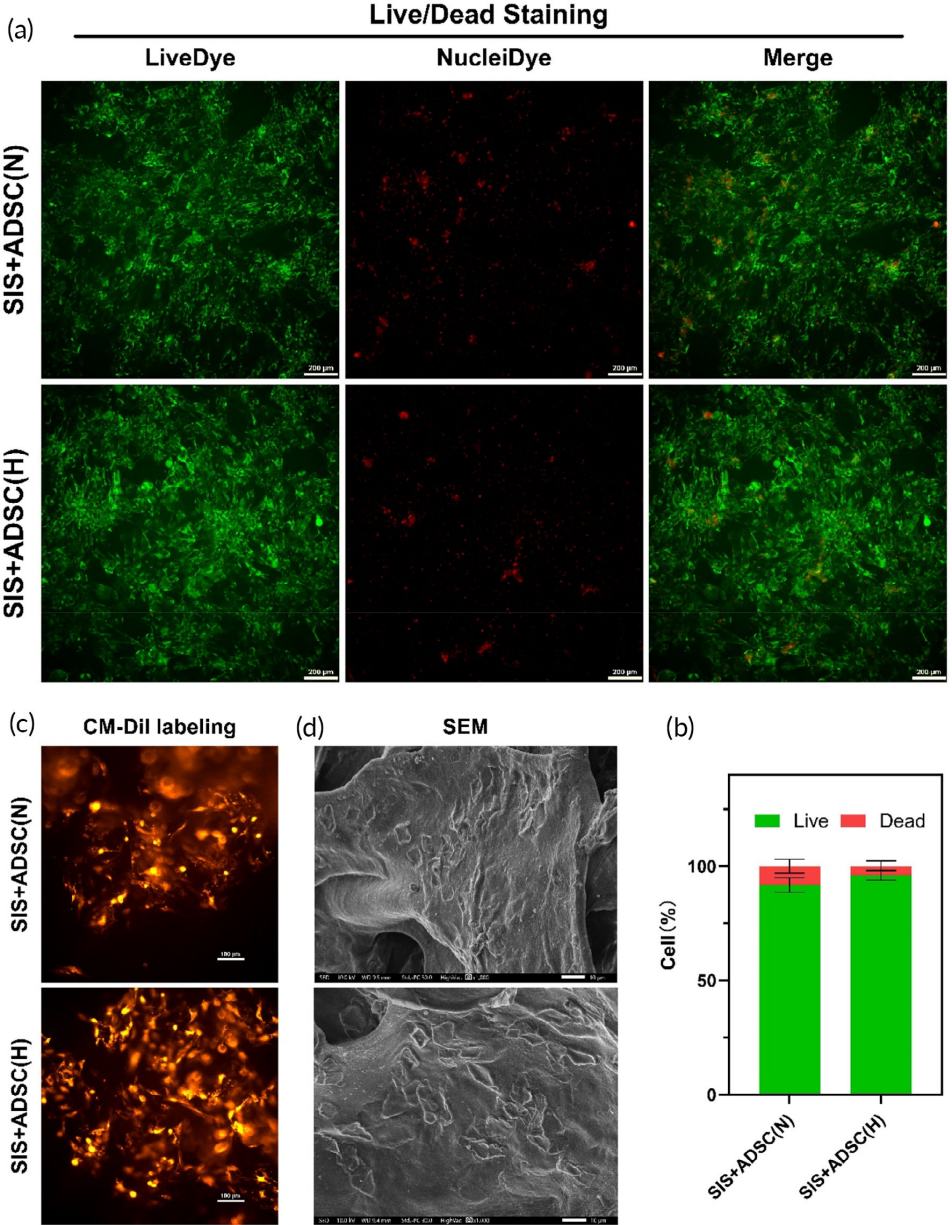
Viability and proliferation of ADSCs seeded on injectable SIS. (A, B) Live/dead staining of the SIS + ADSC composites cultured in normoxia (i.e., the SIS + ADSC(N) group) or preconditioned in hypoxia (i.e., the SIS + ADSC (H) group). Green fluorescence, live cells; red fluorescence, dead cells (scale bar, 200 μm); *p* > 0.05. (C) CM‐Dil labeled ADSCs combined with injectable SIS (Scale bar, 100 μm). (D) SEM observation of the SIS + ADSC composites (Scale bar, 10 μm).

After 24 h of seeding, both SIS + ADSC (N) and SIS + ADSC (H) groups demonstrated high cell viability (Figure 3a,b). Observations from inverted fluorescence microscopy (Figure 3c) and scanning electron microscopy (Figure 3d) revealed that cells were evenly distributed and exhibited diverse morphologies.

**FIGURE 4:**
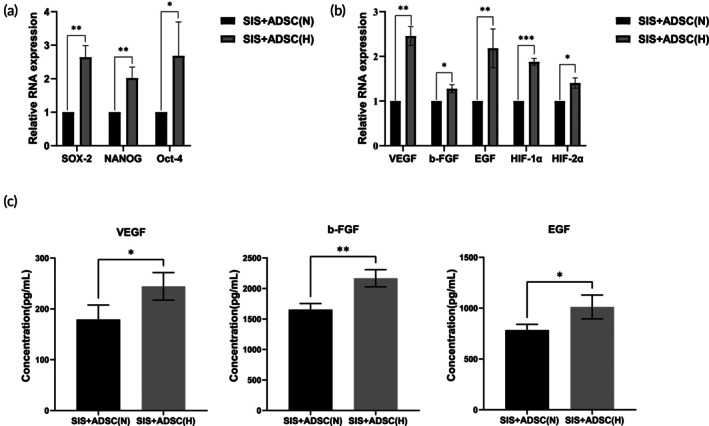
Gene expression and growth factor secretion of the SIS + ADSC composites. (A, B) Gene expression of the SIS + ADSC composites. (C) Growth factor secretion of the SIS + ADSC composites. The values presented are the means ± SEM. *p < 0.05, **p < 0.01, ***p < 0.001.

To investigate the effect of hypoxia preconditioning on the expression of genes associated with stemness, angiogenesis, and epithelial repair in ADSCs, RT‐PCR was conducted to evaluate the levels of NANOG, SOX‐2, Oct‐4, HIF‐1α, HIF‐2α, VEGF, bFGF, and EGF in the SIS + ADSC (N) and SIS + ADSC (H) groups. The findings revealed that hypoxia preconditioning notably enhanced the expression of stem cell‐related genes such as NANOG, SOX‐2, and Oct‐4 (Figure 4a). Furthermore, the hypoxia‐preconditioned group exhibited significantly higher expression of hypoxia‐inducible factor HIF‐1α, angiogenesis‐related gene VEGF, and epithelial repair‐associated genes bFGF and EGF (Figure 4b). In addition, the SIS + ADSC (H) group released higher amounts of growth factors, including VEGF, bFGF, and EGF, compared with the SIS + ADSC (N) group (Figure 4c).

### 3.3 Hypoxia preconditioning enhanced the therapeutic effects of SIS + ADSC composites in OA rats

**FIGURE 5:**
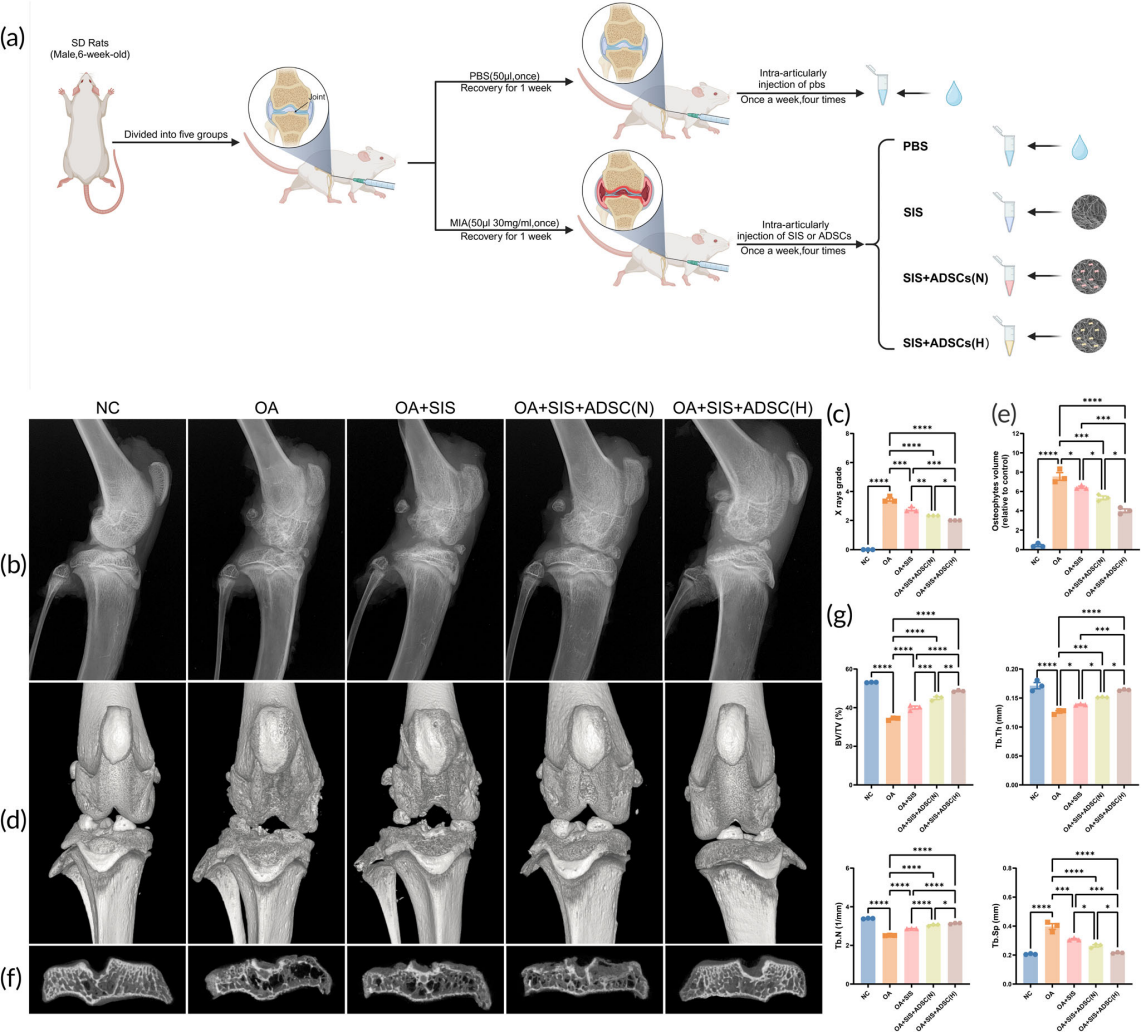
The SIS + ADSCs composite accelerated cartilage repair of OA. (A) Schematic description of the animal model used in this study. (B,C) The x‐ray images of rat knee and x‐ray grade, *n* = 3. (D, E) Micro‐CT images of rat knee and osteophytes volume, *n* = 3. (F, G) Micro‐CT images of subchondral bone and relevant parameters, including total bone volume/total tissue volume (BV/TV), trabecular number (Tb.N), thickness (Tb.Th), and separation (Tb.Sp), *n* = 3. The values presented are the means ± SEM. *p < 0.05, **p < 0.01, ***p < 0.001, ****p < 0.0001.

To evaluate the effects of injectable SIS and ADSCs on OA, an OA model was induced in rats through intra‐articular injection of MIA. Following OA modeling, the SIS, SIS + ADSC (N), and SIS + ADSC (H) groups were administered intra‐articular injections of 50 μL 10 wt% SIS, SIS + ADSC (N), and SIS + ADSC (H), respectively, once weekly for 4 weeks (Figure 5a). The x‐ray imaging revealed significantly greater OA severity in the OA group compared with the NC group, whereas the SIS + ADSC (H) group exhibited a marked reduction in OA severity compared with the OA group (Figure 5b,c). Similarly, micro‐CT scans of knee joints and reconstructed tibial subchondral bone showed that the SIS + ADSC (H) group exhibited reduced osteophyte formation, increased BV/TV, Tb.Th, Tb.N, and Tb.Sp compared with the OA group (Figure 5d–g). These results suggest that SIS + ADSC (H) effectively inhibits bone destruction and joint degeneration.

**FIGURE 6:**
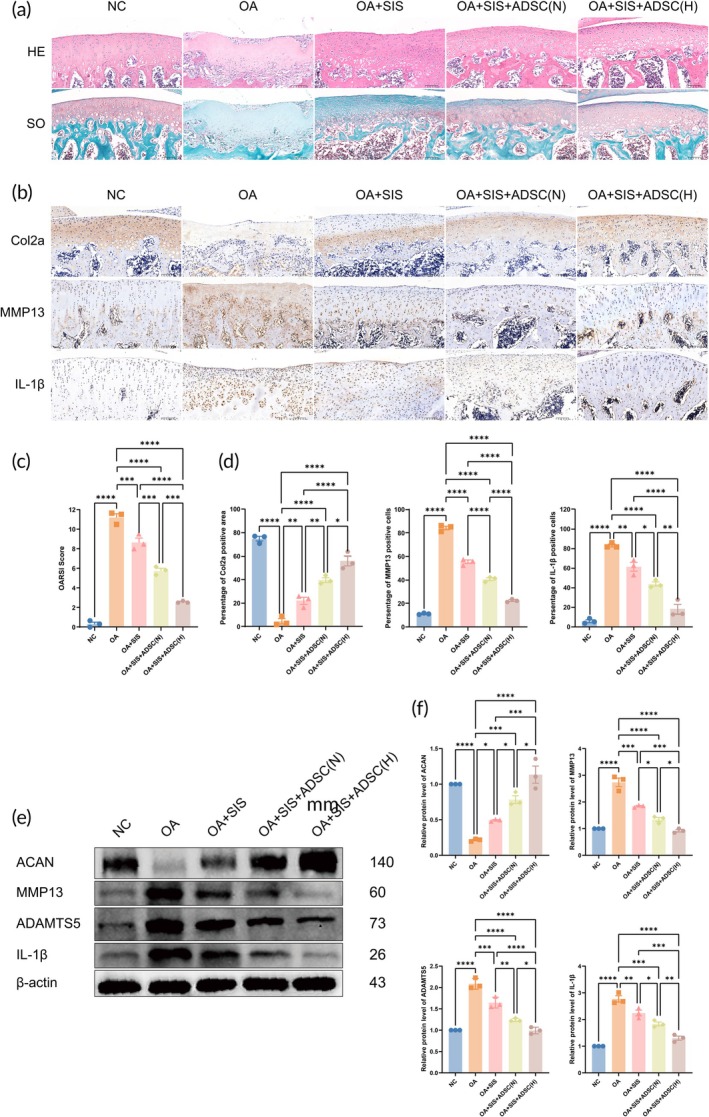
Effect of SIS + ADSCs composite on MIA‐induced rat OA model. (A, C) Morphology of cartilage shown in HE staining and Safranin O staining and the Osteoarthritis Research Society International (OASRI) score, *n* = 3. (B, D) Immunohistochemical staining assay of Col2a, MMP13, and IL‐1β in the cartilage, *n* = 3. (E, F) The protein levels of ACAN, MMP13, and ADAMTS5, IL‐1β were presented by Western blotting, *n* = 3. Full‐length blots/gels are presented in Additional file 2. The values presented are the means ± SEM. *p < 0.05, **p < 0.01, ***p < 0.001, ****p < 0.0001.

Histopathological staining after modeling revealed severe cartilage degeneration, chondrocyte apoptosis and loss, extensive collagen degradation, and matrix disorganization, all characteristic of a typical OA phenotype. Compared with the NC group, the OA model group exhibited significantly lower chondrocyte counts and glycosaminoglycan content (a major cartilage matrix component), increased chondrocyte apoptosis (Figure 6a), and a significantly higher OARSI score (*p* < 0.0001) (Figure 6c). Intra‐articular injections of SIS and ADSCs increased chondrocyte counts, decreased apoptosis, enhanced the structural integrity of articular cartilage, promoted glycosaminoglycan synthesis, and reduced OARSI scores. These effects were more pronounced in the SIS + ADSC (H) group compared with the SIS + ADSC (N) and SIS groups (Figure 6c). Immunohistochemical analysis revealed significant changes in the expression of Col2a (a cartilage matrix component), MMP13 (a catabolic marker), and IL‐1β (an inflammatory marker) in the OA group compared with the NC group (all *p* < 0.0001). SIS and ADSC treatments significantly restored the expression of these markers to near‐normal levels (Figure 6b,d). Notably, the SIS + ADSC (H) group demonstrated greater improvements in immunohistochemical marker expression compared with the SIS + ADSC (N) and SIS groups. Western blot analysis further confirmed that the SIS + ADSC (H) group significantly reversed the altered expression of ACAN, MMP13, ADAMTS5, and IL‐1β proteins in chondrocytes of the OA model group (Figure 6e,f). Collectively, these findings indicate that hypoxia‐preconditioned ADSCs combined with injectable SIS effectively reverse MIA‐induced chondrocyte protein expression abnormalities and offer significant therapeutic benefits in OA rats.

### 3.4 SIS + ADSC (H)‐CM suppresses ECM degradation and inflammation while promoting ECM synthesis in IL‐1β‐treated chondrocytes

**FIGURE 7:**
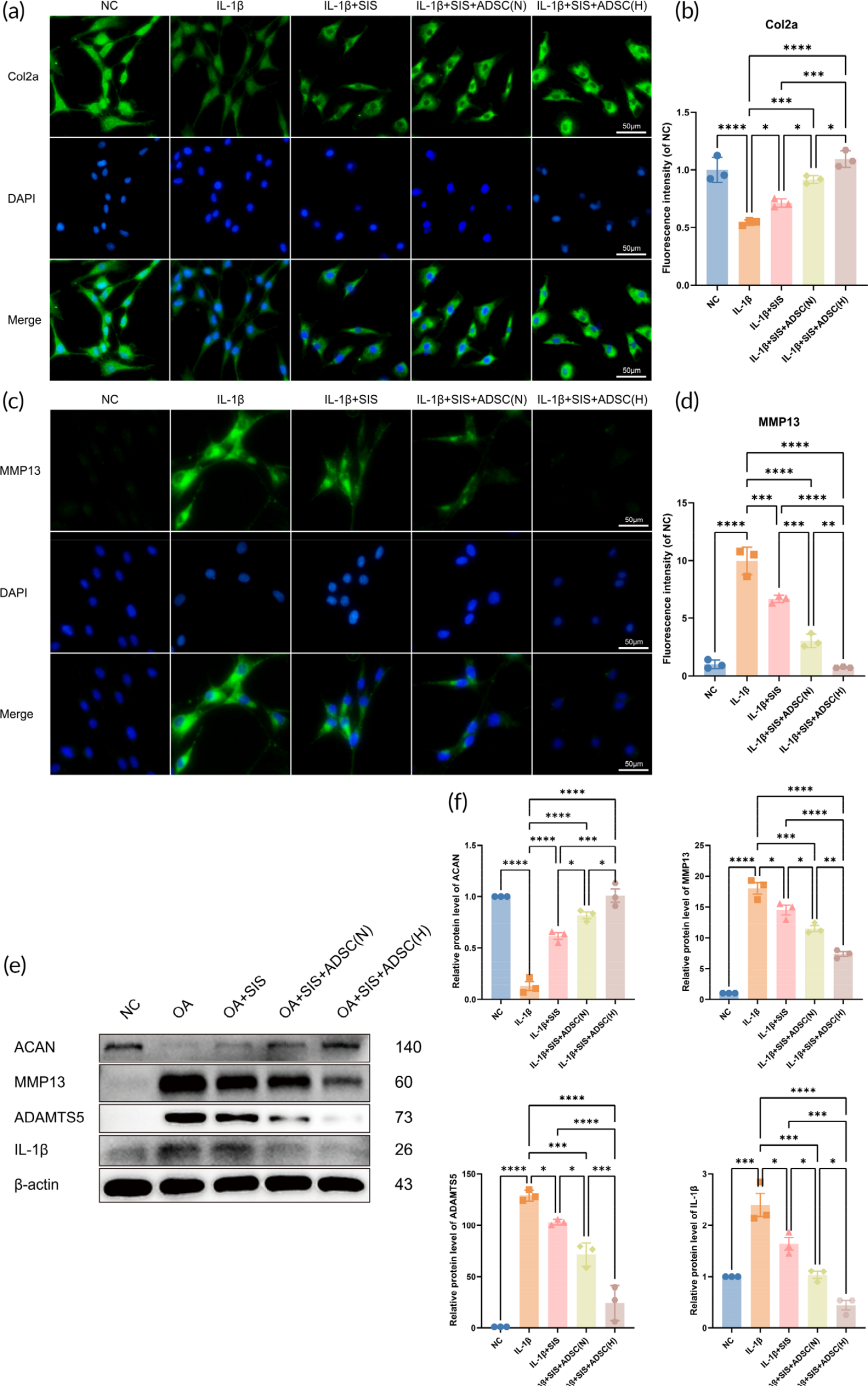
Effects of SIS + ADSC‐CM on extracellular matrix synthesis and degradation in IL1β‐induced chondrocytes. All image sizes and magnifications are consistent. (A–D) The expression of Col2a and MMP13 was detected by immunofluorescence using rat chondrocytes, *n* = 3. (E, F) The protein levels of ACAN, MMP13, and ADAMTS5, IL‐1β were presented by Western blotting, *n* = 3. Full‐length blots/gels are presented in Additional file 2. The values presented are the means ± SEM. *p < 0.05, **p < 0.01, ***p < 0.001, ****p < 0.0001.

To evaluate the effects of SIS and ADSCs on IL‐1β‐stimulated chondrocytes, the expression of matrix synthesis components (Aggrecan, ACAN), matrix degradation markers (MMP13 and ADAMTS5), and the inflammatory cytokine interleukin‐1β (IL‐1β) was analyzed. Western blot analysis (Figure 7e,f) showed that IL‐1β stimulation increased the expression of matrix degradation markers and inflammatory factors while reducing matrix synthesis component levels. SIS and ADSC treatments alleviated IL‐1β‐induced increases in MMP13, ADAMTS5, and IL‐1β levels and restored ACAN levels, with more significant improvements observed in the SIS + ADSC (H) group compared with the SIS + ADSC (N) and SIS groups. Immunofluorescence staining confirmed that SIS + ADSC (H)‐CM suppressed ECM degradation and promoted ECM synthesis in IL‐1β‐stimulated chondrocytes (Figure 7a–d).

## 4 DISCUSSION

Stem cell‐based therapies offer significant promise for cartilage repair in arthritis treatment.^50^ In this study, we designed a minimally invasive, efficient, and convenient biomaterial composite—termed SIS + ADSC—by combining ADSCs with injectable SIS, a biomaterial commonly used for soft tissue repair. The impact of hypoxia preconditioning on the cartilage repair potential of this composite was evaluated through in vitro and in vivo experiments. Our findings revealed that the SIS + ADSC composite achieved significantly better cartilage repair outcomes compared with SIS alone. Notably, hypoxia preconditioning markedly improved the cartilage repair potential of the SIS + ADSC composite. Experimental data showed that hypoxia preconditioning elevated the expression of growth factors, hypoxia‐inducible factors, and stem cell‐related genes (Figure 4), enhancing angiogenesis and glycolysis to support cartilage repair.^40^, ^51^

While stem cells hold promise for cartilage regeneration, their therapeutic potential is often limited by low survival rates after transplantation.^52^, ^53^ SIS, a naturally derived scaffold, offers excellent attachment points and a favorable microenvironment for cells, attributed to its high collagen and glycosaminoglycan content. SIS also incorporates key growth factors (GFs), including VEGF, FGF‐2, and TGF‐β, which bind to proteins and are gradually released during SIS degradation, thereby stimulating and guiding endogenous cell migration to the injury site to support cartilage repair.^54^ The three‐dimensional porous structure of the SIS scaffold enhances nutrient and waste exchange, while its mechanical properties offer elastic support similar to that of articular cartilage.^55^, ^56^ Accordingly, we developed a three‐dimensional injectable SIS that supports ADSC growth and provides enhanced operability. This system enables minimally invasive intra‐articular injection of ADSCs to enhance cartilage repair in arthritis. The biocompatibility of ADSCs with injectable SIS was confirmed using live/dead staining, scanning electron microscopy, and CM‐Dil labeling of ADSCs (Figure 3).^57^

It is now widely acknowledged that stem cell‐based therapies primarily rely on paracrine effects for repair, rather than direct cell differentiation.^19^, ^58^ Consequently, the therapeutic potential of exosomes and cytokines secreted by mesenchymal stem cells has been investigated for cartilage repair, yielding promising results.^59^, ^60^, ^61^, ^62^ Hypoxia‐preconditioned stem cells are believed to enhance their therapeutic potential by boosting paracrine activity. Studies have shown that hypoxia‐preconditioned adipose‐derived MSCs promote cartilage regeneration mainly through a substantial increase in growth factor secretion.^31^, ^32^, ^57^ Hypoxia‐preconditioned ADSCs show significant upregulation of HIF‐1α, which promotes VEGF expression. VEGF also activates the PI3K/Akt signaling pathway, boosting the anti‐apoptotic capacity of ADSCs and increasing their survival at the injury site.^33^, ^63^ Hypoxia preconditioning further enhances the expression of stemness markers, such as Oct‐4 and NANOG, which preserve ADSC multipotency and stimulate cell proliferation, significantly improving their survival in cartilage injury regions.^63^, ^64^ Consistent with these findings, this study demonstrated that hypoxia preconditioning of the SIS + ADSC composite upregulated stem cell‐related genes and growth factors. Notably, in vivo results showed that the SIS + ADSC (H) group achieved significantly better cartilage repair outcomes compared with the other groups.

This study employed an MIA‐induced rat model of osteoarthritis to assess the in vivo anti‐osteoarthritis and cartilage repair effects of the SIS + ADSC composite and used an IL‐1β‐induced chondrocyte model to investigate its cellular and molecular mechanisms. In vivo results demonstrated that the SIS + ADSC composite exerts multifaceted effects on OA by protecting chondrocytes and enhancing cartilage repair (Figures 5 and 6). Moreover, SIS + ADSC (H) showed significantly enhanced anti‐OA effects compared with SIS and SIS + ADSC (N) (*p* < 0.05), highlighting its potential for OA treatment. These protective and reparative effects on chondrocytes were mediated through restoring anabolic activity (upregulating Aggrecan), suppressing catabolic activity (downregulating ADAMTS5 and MMP13), and reducing inflammation (downregulating IL‐1β) (Figure 6). In vitro experiments showed that the conditioned medium from the SIS + ADSC composite regulates anabolic and catabolic metabolism as well as inflammation in chondrocytes, aligning with the in vivo findings. Furthermore, SIS + ADSC (H)‐CM influenced chondrocytes via paracrine mechanisms (Figures 4 and 7). The key innovations and highlights of this study include: (1) development of a minimally invasive, efficient, and convenient biomaterial composite, with its anti‐OA efficacy and safety validated through in vitro and in vivo studies; (2) investigation of therapeutic differences between hypoxia‐preconditioned and normoxia‐cultured ADSCs; and (3) exploration of the paracrine effects of ADSCs using the SIS + ADSC composite's conditioned medium.

This study has several limitations that should be noted. While this study confirmed the short‐term cartilage repair benefits of the SIS + ADSC complex, its long‐term effects and potential side effects have not been fully evaluated. Additionally, hypoxic preconditioning greatly improved the repair potential of ADSCs, though the underlying mechanisms are still unclear. The interaction between ADSCs and the damaged cartilage environment during repair needs further investigation. In light of these issues, further in‐depth studies will be conducted in the future.

## 5 CONCLUSION

This study confirms that hypoxia preconditioning significantly enhances the cartilage repair potential of ADSCs in arthritis. Hypoxia preconditioning markedly boosted the secretion of growth factors. Compared with the SIS + ADSC (N) group, the SIS + ADSC (H) group exhibited superior protective and reparative effects on chondrocytes by enhancing anabolic activity, reducing catabolism, and suppressing inflammation. In conclusion, hypoxia preconditioning provides a straightforward and effective approach to boost the cartilage repair capacity of ADSCs. The intra‐articular injection of the SIS + ADSC composite represents a convenient and effective strategy for cartilage repair in osteoarthritis treatment.

## AUTHOR CONTRIBUTIONS

**Kun Yu:** Writing—original draft; software; project administration; methodology; investigation; formal analysis; data curation. **Liang Ma:** Project administration; methodology; formal analysis; data curation. **Pengkun Han:** Project administration; methodology. **Yinshen Liu:** Validation; data curation; conceptualization. **Longfei Zou:** Visualization; validation; supervision; methodology; conceptualization. **Sen Wang:** Software; methodology; data curation. **Jiesi Hu:** Project administration; methodology. **Kai Zhong:** Project administration; methodology. **Jiaqiang Liu:** Validation; supervision. **Bo Guo:** Validation; supervision; data curation. **Jie Zou:** Methodology. **Houyin Shi:** Visualization; validation; supervision; resources; funding acquisition. **Xing Guo:** Writing—review and editing; visualization; funding acquisition; data curation; conceptualization. **Meiyun Tan:** Writing—review and editing; supervision; funding acquisition; conceptualization.

## FUNDING INFORMATION

This work was supported by Sichuan Science and Technology Program (Grant No: 2022YFS0609).

## CONFLICT OF INTEREST STATEMENT

The authors declare that they have no known competing financial interests or personal relationships that could have appeared to influence the work reported in this paper.

## ETHICS STATEMENT

The approved project title is “The Role and Mechanism of Hypoxia‐Preconditioned Adipose‐Derived Mesenchymal Stem Cells Combined with Injectable Porcine Small Intestinal Submucosa in Cartilage Defect Repair for Knee Osteoarthritis.” All animal procedures were approved by the Animal Ethics Committee of Southwest Medical University (Approval No. 20221104‐015; Approval Date: 04/11/2022).

## Supporting information


**Data S1.** Supporting Information.

**Data S2.** Supporting Information.

## Data Availability

The data that support the findings of this study are available from the corresponding author upon reasonable request.
