# Supplementary material for: Hypoxia‐preconditioned adipose‐derived stem cells with injectable small intestinal submucosa for enhanced cartilage repair in osteoarthritis
Source: Bioeng Transl Med. 2026 Feb 2;11(3):e70116. doi: 10.1002/btm2.70116 (PMC13247430; doi:10.1002/btm2.70116)
Supplement: Supplementary file 1 — Data S1. Supporting Information. [file BTM2-11-e70116-s002.zip › Additional file 1/PCR primers.pdf]

| Gene | Forward primer | Reverse primer |
| --- | --- | --- |
| β-Actin | 5′-CCCATCTATGAGGGTTACGC-3′ | 5′-TTTAATGTCACGCACGATTTC-3′ |
| SOX-2 | 5′-GAACTAGACTCCGGGCGATG-3′ | 5′-CCCAGCAAGAACCCTTTCCT-3′ |
| NANOG | 5′-TGTTAACGATGGGCTTCGGA-3′ | 5′-GTAGGGTGGGTGTGTGAGAC-3′ |
| Oct-4 | 5′-GGCTTCAGACTTCGCCTTCT-3′ | 5′-GCCATCCCTCCACAGAACTC-3′ |
| VEGF | 5′-TGGTGGACATCTTCCAGGAGTACC-3′ | 5′-ATCCGCATGATCTGCATAGTGACG-3′ |
| b-FGF | 5′-CCACACGTCAAACTACAGCT-3′ | 5′-AACACACTTAGAAGCCAGCA-3′ |
| EGF | 5′-CCATGCTGTTCTCGCTCACCTTC-3′ | 5′-GTTCCTGGTCTGCTGTGCTGTG-3′ |
| HIF-1a | 5′-CTCCCTTTTTCAAGCAGCAG-3′ | 5′-GCTCCATTCCATCCTGTTCA -3′ |
| HIF-2a | 5′-CTTCCCAGCCACCATCTACC-3′ | 5′-GTCGACTTGCCACTCCTGAC -3′ |
