# Supplementary figures and images for: Hypoxia‐preconditioned adipose‐derived stem cells with injectable small intestinal submucosa for enhanced cartilage repair in osteoarthritis
Source: Bioeng Transl Med. 2026 Feb 2;11(3):e70116. doi: 10.1002/btm2.70116 (PMC13247430; doi:10.1002/btm2.70116)

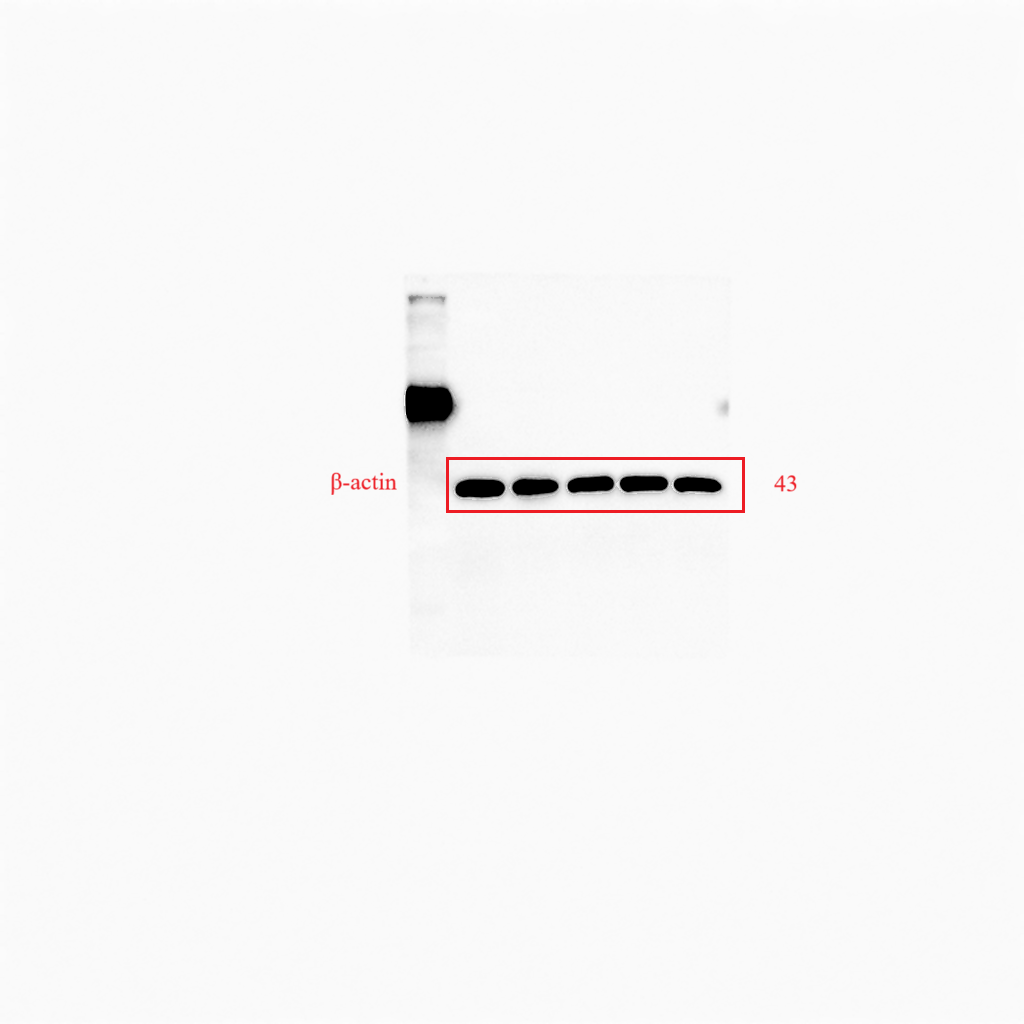

Supplement: Supplementary file 2 — Data S2. Supporting Information. [file BTM2-11-e70116-s001.zip › Additional file 2/Cell WB Supplementary Figure/ACAN/Fig.7 WB-ACNA(β-actin).Tif]

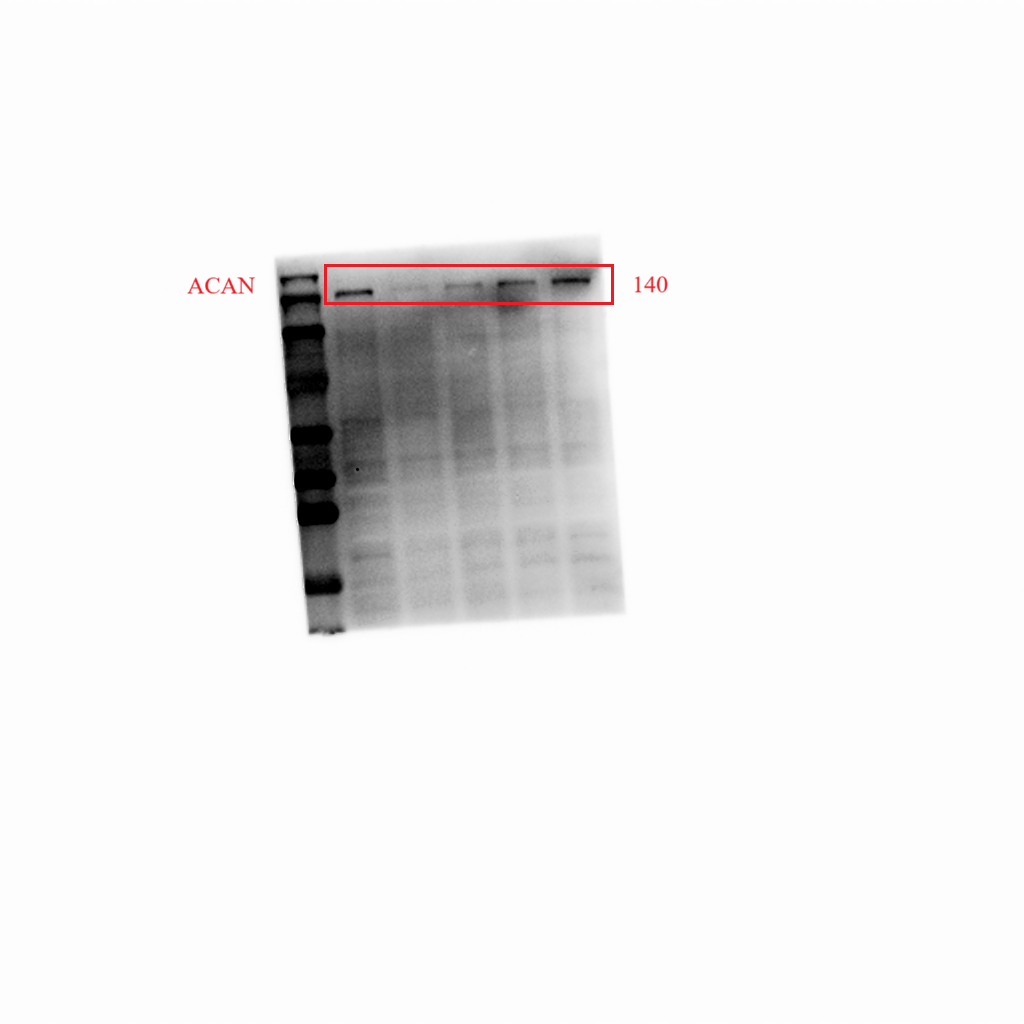

Supplement: Supplementary file 2 — Data S2. Supporting Information. [file BTM2-11-e70116-s001.zip › Additional file 2/Cell WB Supplementary Figure/ACAN/Fig.7 WB-ACNA.Tif]

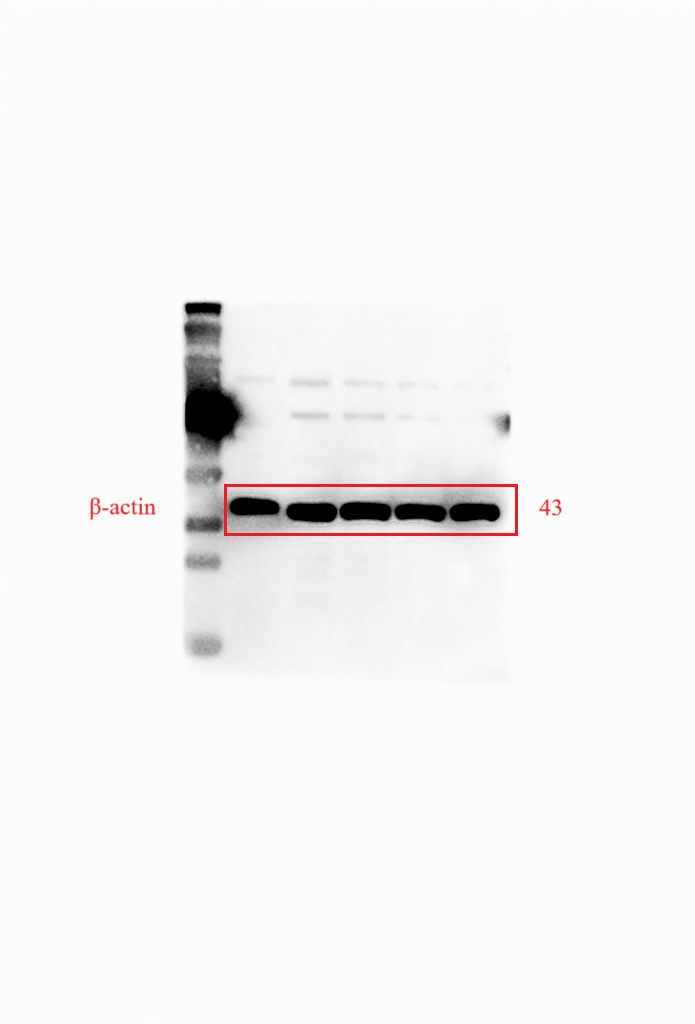

Supplement: Supplementary file 2 — Data S2. Supporting Information. [file BTM2-11-e70116-s001.zip › Additional file 2/Cell WB Supplementary Figure/ADAMTS5/Fig.7 WB-ADAMTS5(β-actin).Tif]

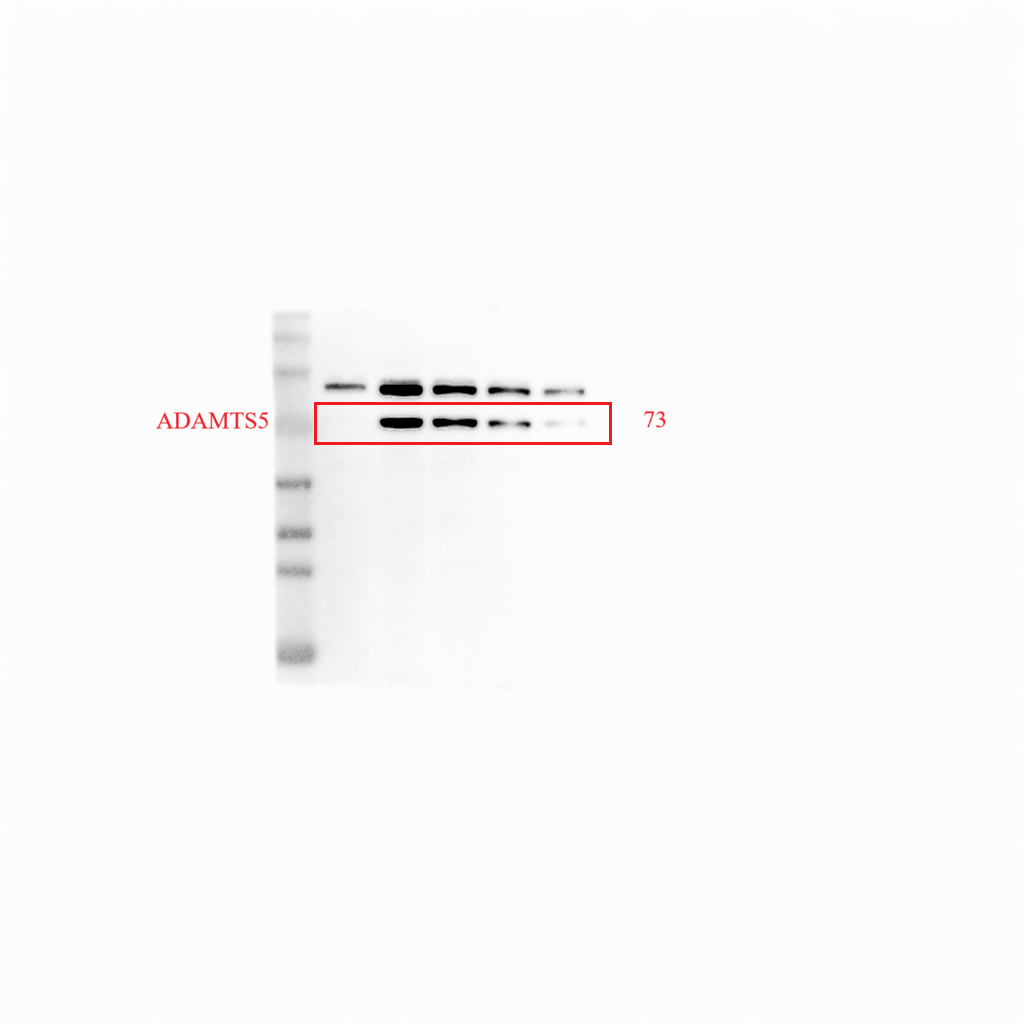

Supplement: Supplementary file 2 — Data S2. Supporting Information. [file BTM2-11-e70116-s001.zip › Additional file 2/Cell WB Supplementary Figure/ADAMTS5/Fig.7 WB-ADAMTS5.Tif]

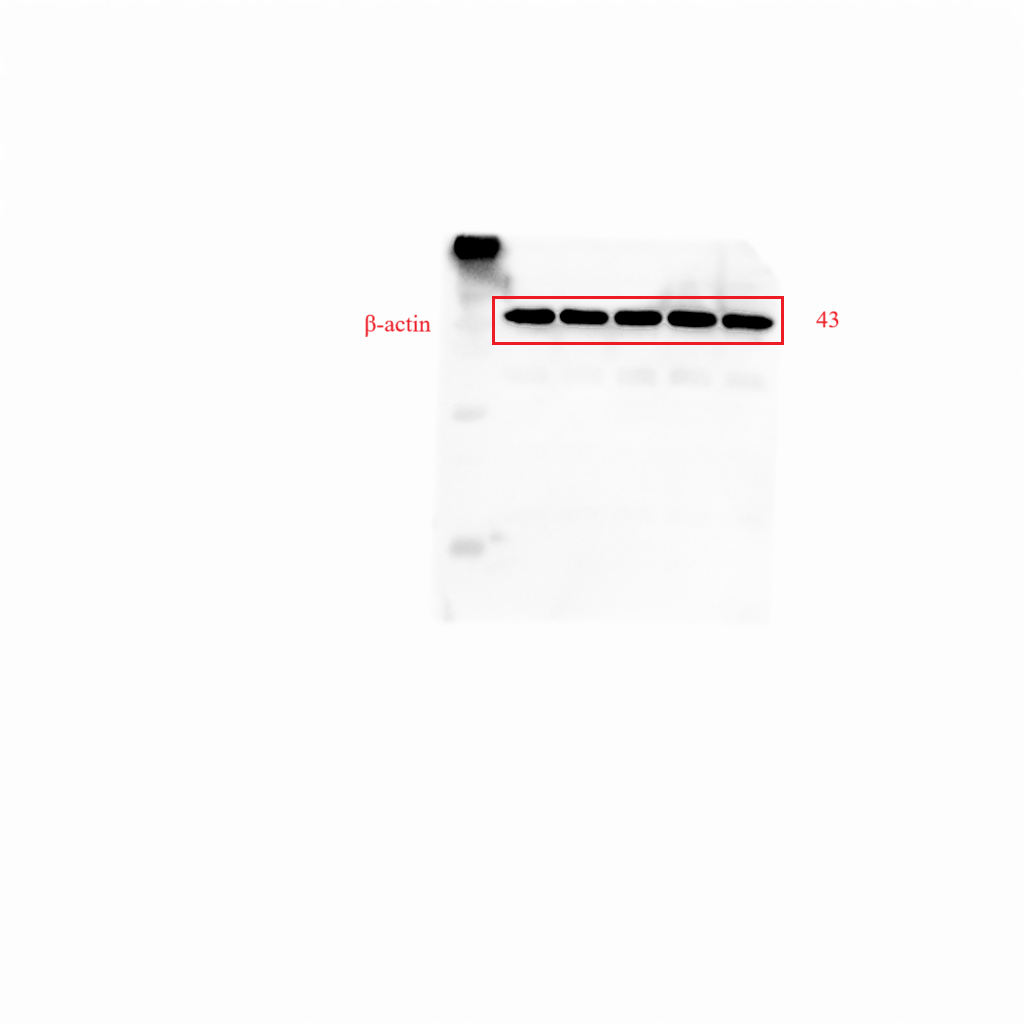

Supplement: Supplementary file 2 — Data S2. Supporting Information. [file BTM2-11-e70116-s001.zip › Additional file 2/Cell WB Supplementary Figure/IL-1β/Fig.7 WB-IL1β(β-actin).Tif]

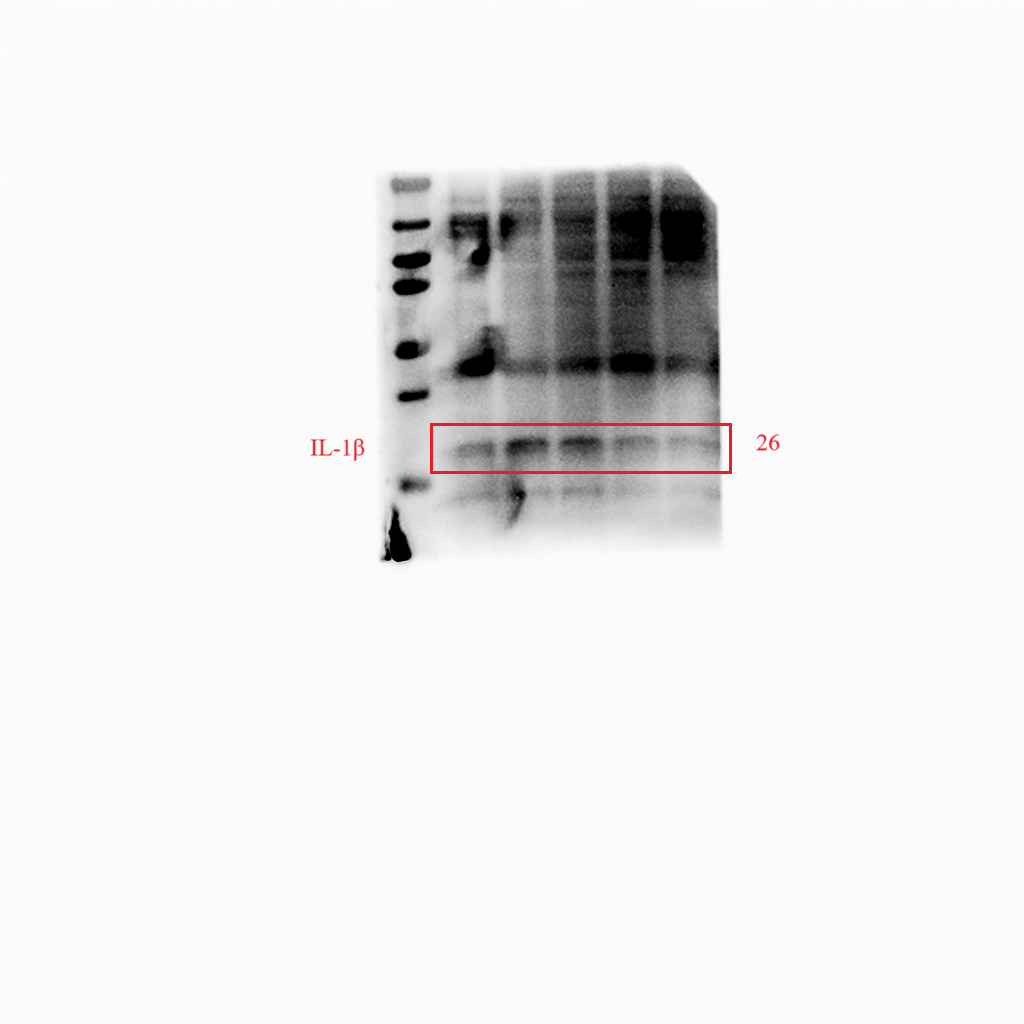

Supplement: Supplementary file 2 — Data S2. Supporting Information. [file BTM2-11-e70116-s001.zip › Additional file 2/Cell WB Supplementary Figure/IL-1β/Fig.7 WB-IL1β.Tif]

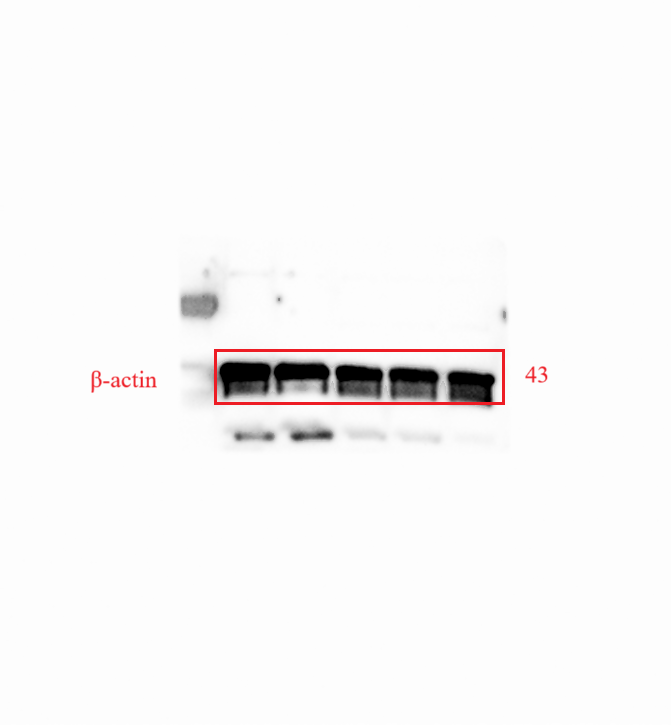

Supplement: Supplementary file 2 — Data S2. Supporting Information. [file BTM2-11-e70116-s001.zip › Additional file 2/Cell WB Supplementary Figure/MMP13/Fig.7 WB-MMP13(β-actin).Tif]

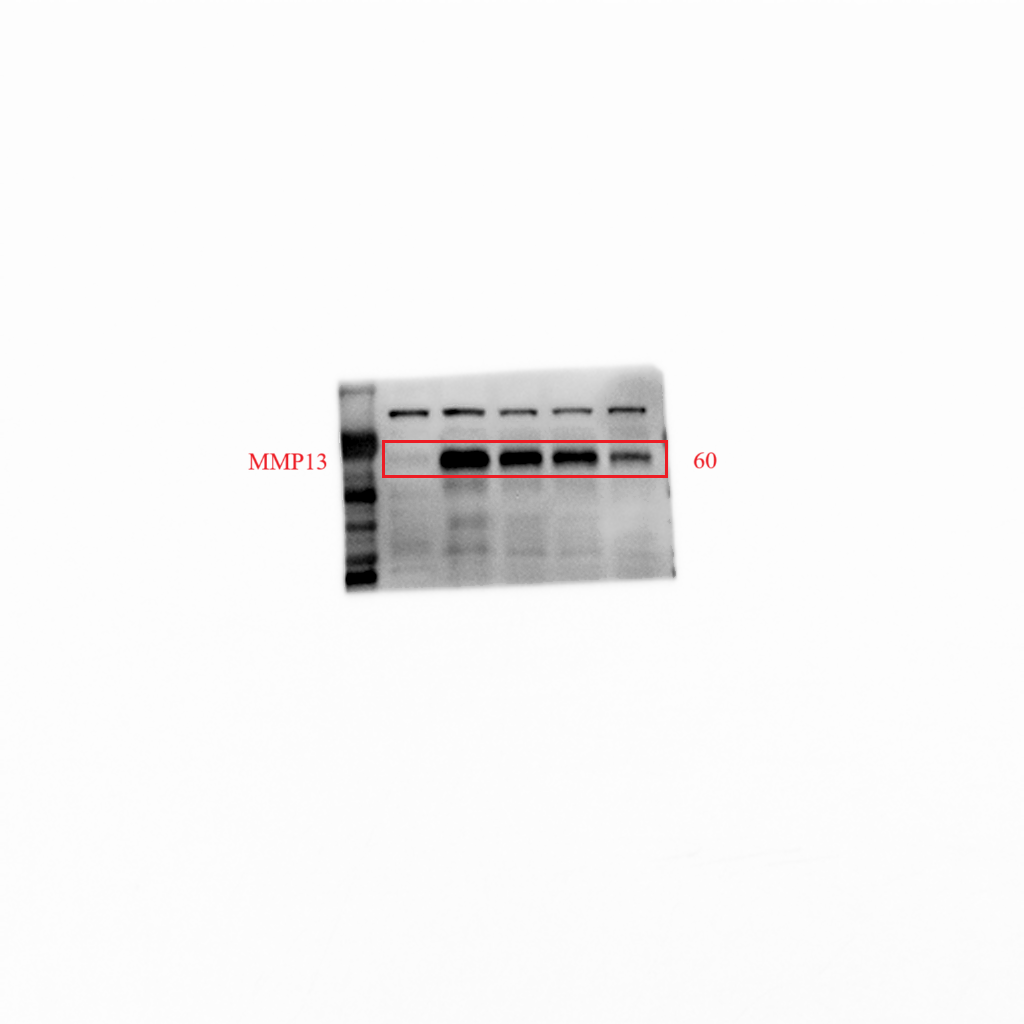

Supplement: Supplementary file 2 — Data S2. Supporting Information. [file BTM2-11-e70116-s001.zip › Additional file 2/Cell WB Supplementary Figure/MMP13/Fig.7 WB-MMP13.Tif]

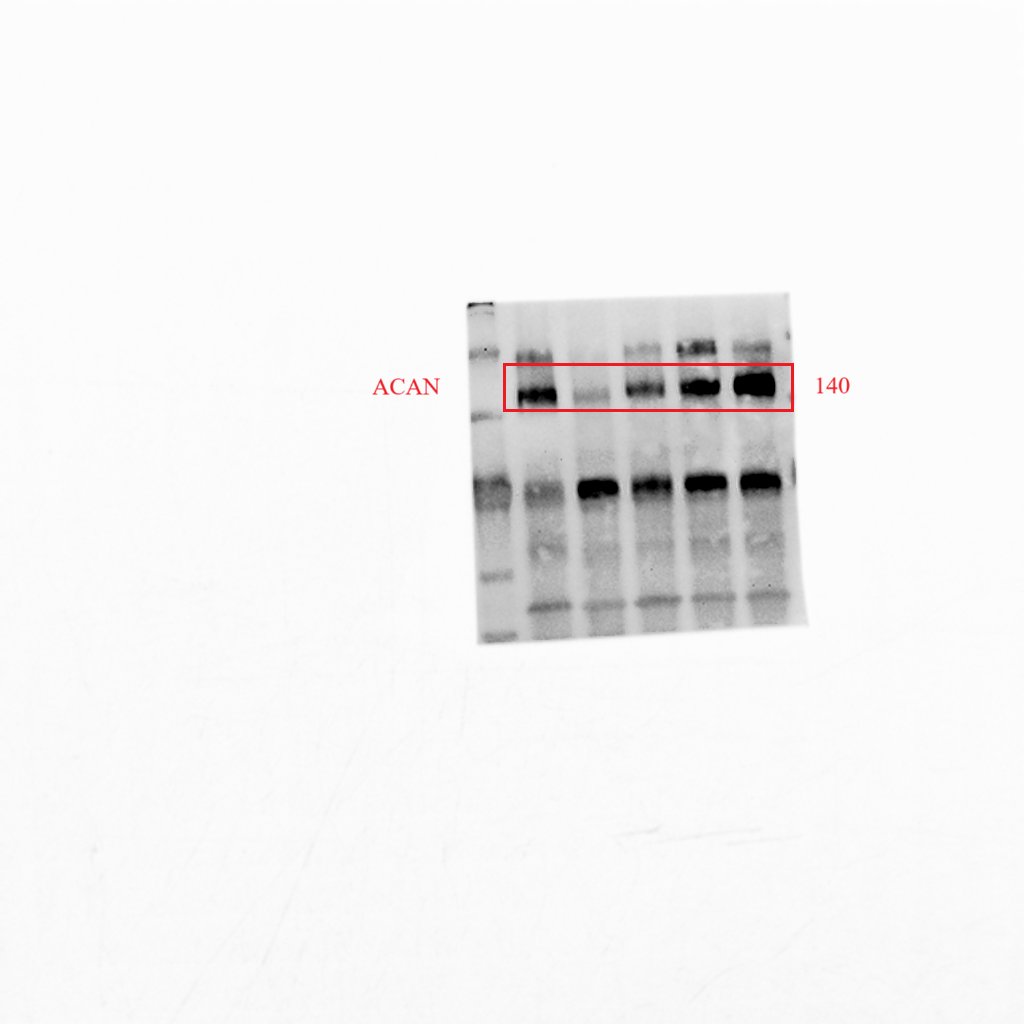

Supplement: Supplementary file 2 — Data S2. Supporting Information. [file BTM2-11-e70116-s001.zip › Additional file 2/Tissue WB Supplementary Figure/ACAN/Fig.6 WB-ACAN.Tif]

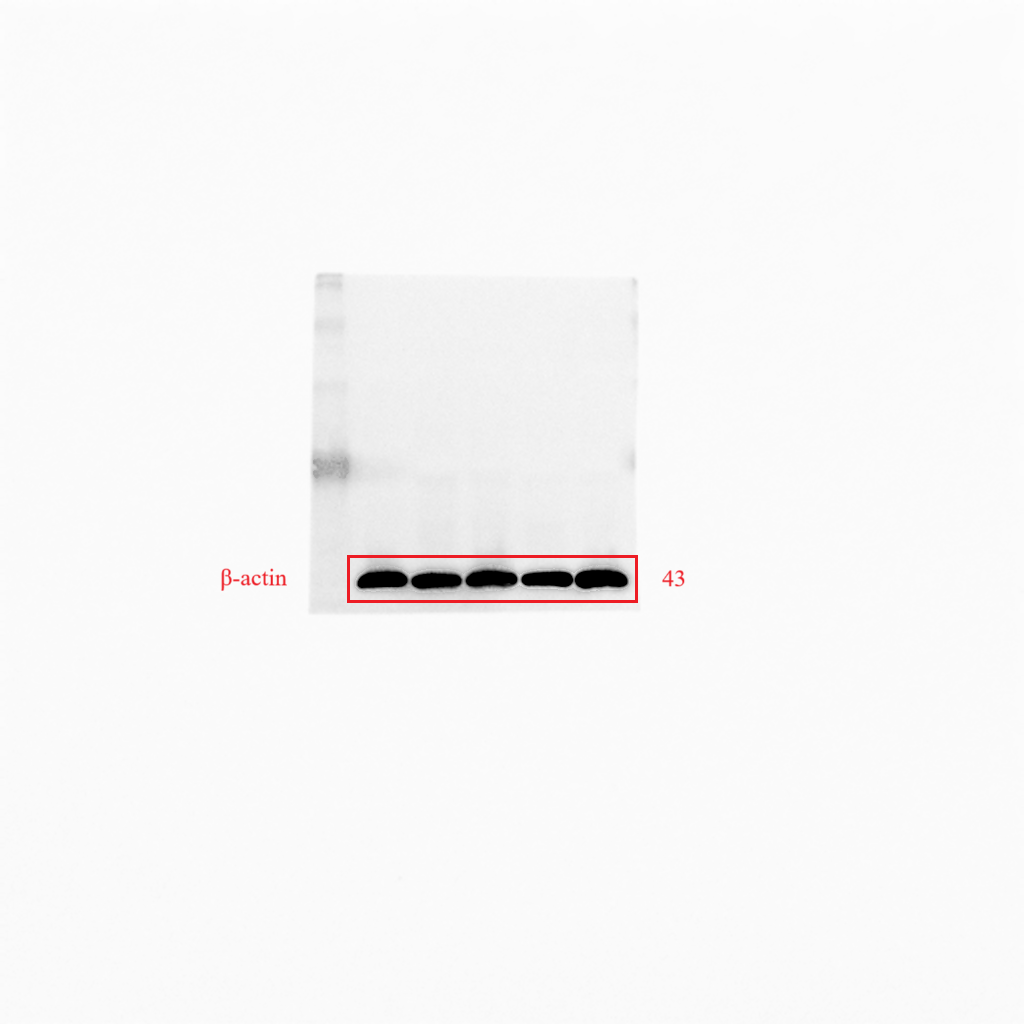

Supplement: Supplementary file 2 — Data S2. Supporting Information. [file BTM2-11-e70116-s001.zip › Additional file 2/Tissue WB Supplementary Figure/ACAN/Fig.6 WB-ACAN(β-actin).Tif]

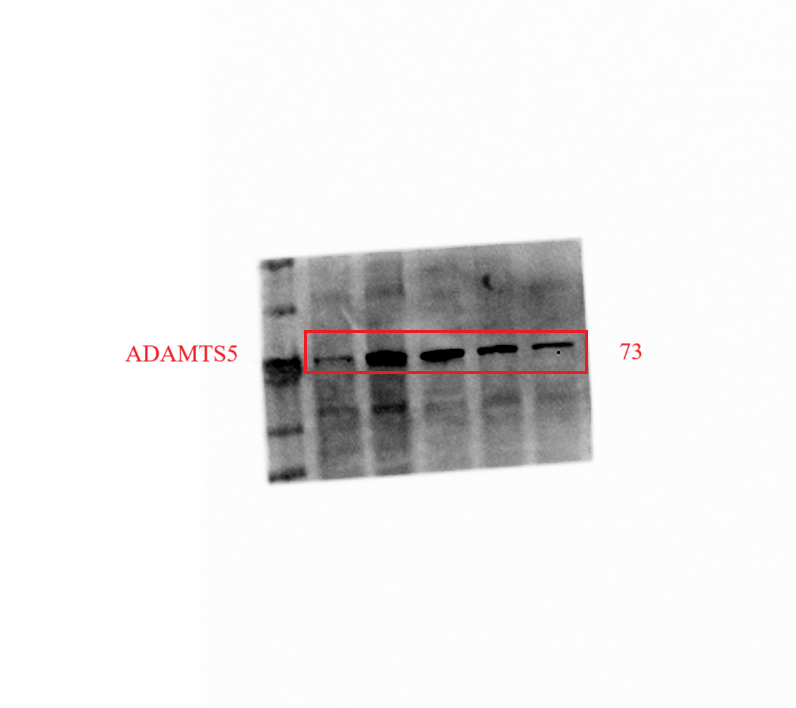

Supplement: Supplementary file 2 — Data S2. Supporting Information. [file BTM2-11-e70116-s001.zip › Additional file 2/Tissue WB Supplementary Figure/ADAMTS5/Fig.6 WB-ADAMTS5.Tif]

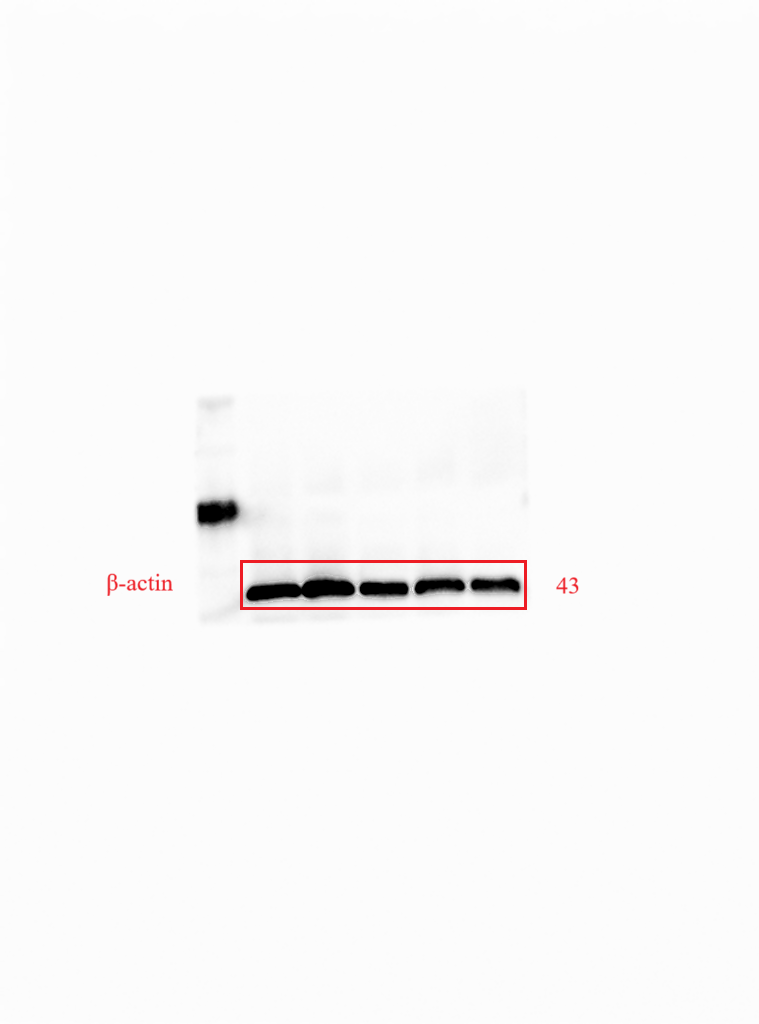

Supplement: Supplementary file 2 — Data S2. Supporting Information. [file BTM2-11-e70116-s001.zip › Additional file 2/Tissue WB Supplementary Figure/ADAMTS5/Fig.6 WB-ADAMTS5(β-actin).Tif]

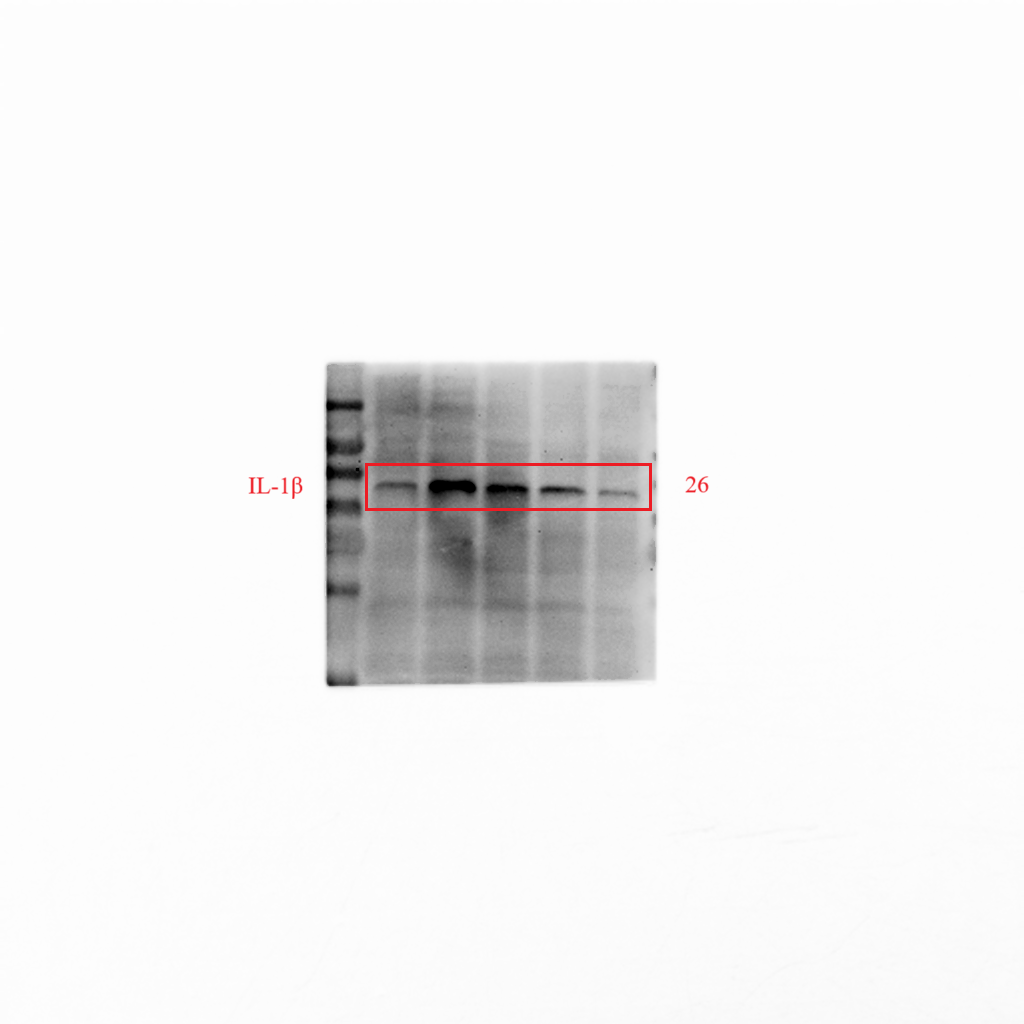

Supplement: Supplementary file 2 — Data S2. Supporting Information. [file BTM2-11-e70116-s001.zip › Additional file 2/Tissue WB Supplementary Figure/IL-1β/Fig.6 WB-IL1β.Tif]

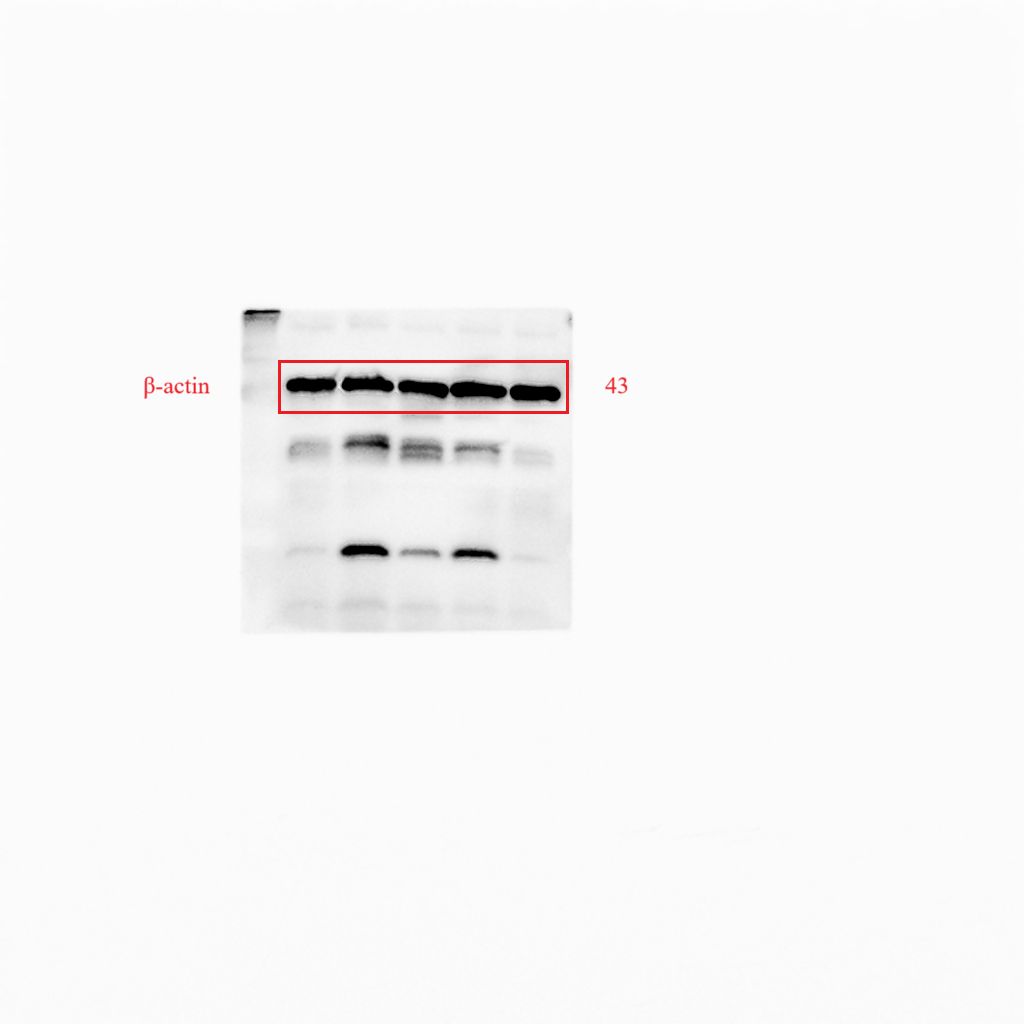

Supplement: Supplementary file 2 — Data S2. Supporting Information. [file BTM2-11-e70116-s001.zip › Additional file 2/Tissue WB Supplementary Figure/IL-1β/Fig.6 WB-IL1β(β-actin).Tif]

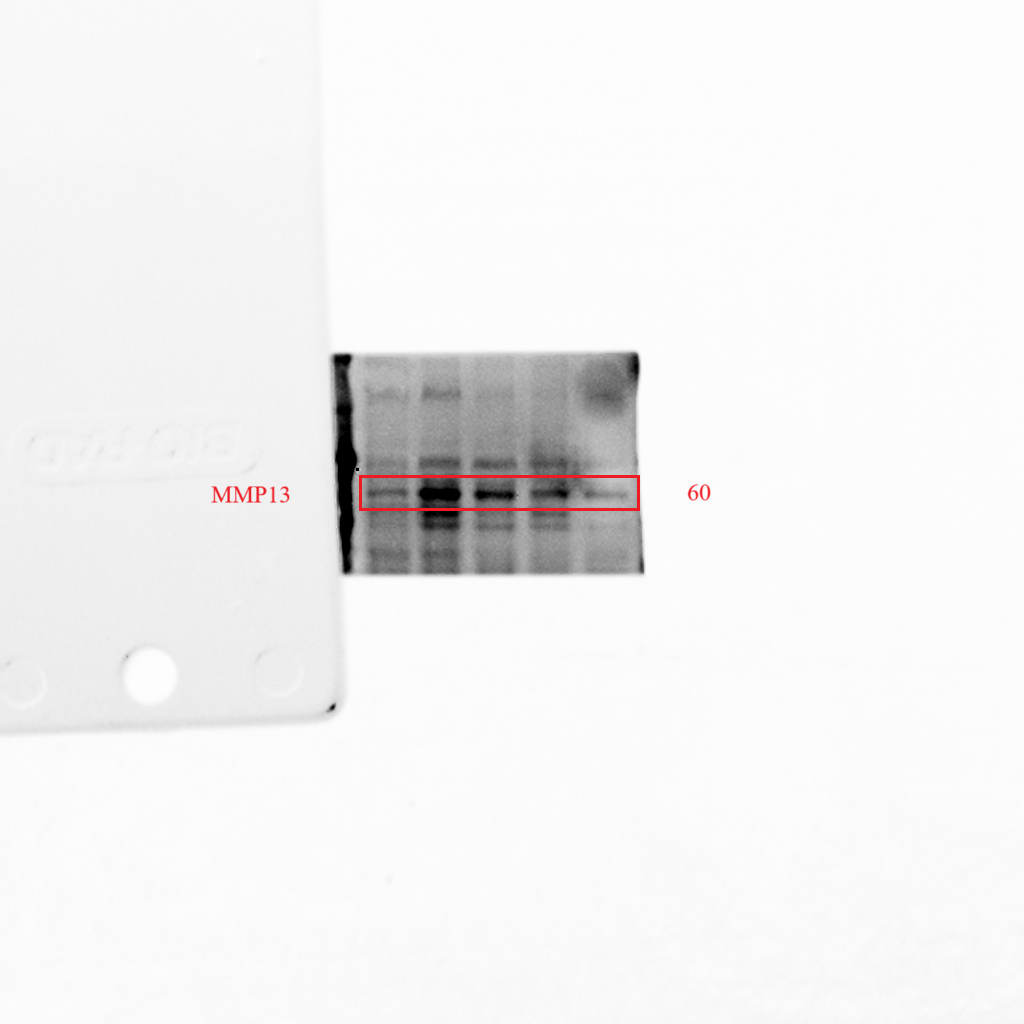

Supplement: Supplementary file 2 — Data S2. Supporting Information. [file BTM2-11-e70116-s001.zip › Additional file 2/Tissue WB Supplementary Figure/MMP13/Fig.6 WB-MMP13.Tif]

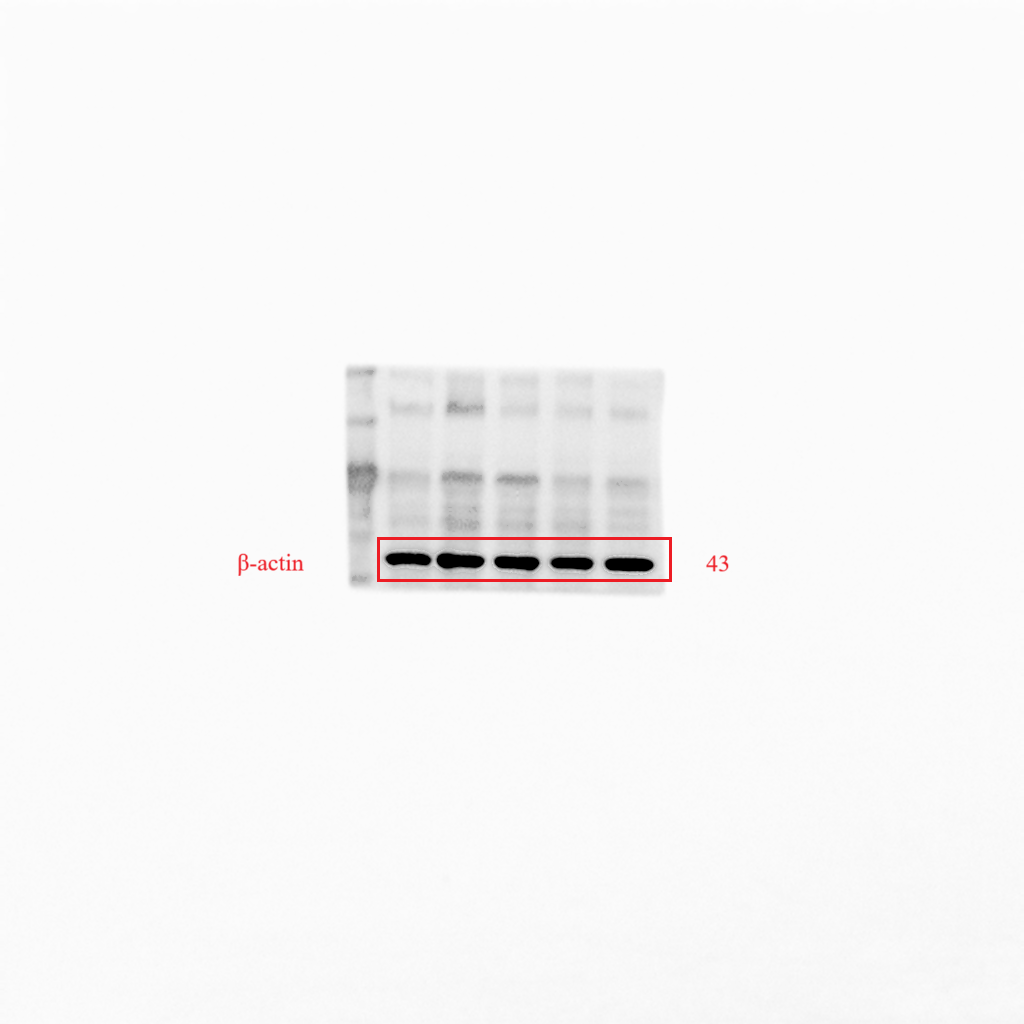

Supplement: Supplementary file 2 — Data S2. Supporting Information. [file BTM2-11-e70116-s001.zip › Additional file 2/Tissue WB Supplementary Figure/MMP13/Fig.6 WB-MMP13(β-actin).Tif]
